# Prognostic Evidence for Artificial Intelligence-Derived PET Tumour-Burden Biomarkers in Cancer: A Systematic Review

**DOI:** 10.3390/diagnostics16213564

**Published:** 2026-10-29

**Authors:** Sang Jun Byun, Yunhwi Hwang, Hae Won Kim, Byungwook Choi

**Affiliations:** 1 Department of Radiation Oncology, Keimyung University Dongsan Hospital, Keimyung University School of Medicine, Daegu 42601, Republic of Korea; kryph@dsmc.co.kr; 2 Division of Hematology-Oncology, Department of Internal Medicine, Daegu Catholic University Medical Center, Daegu 42472, Republic of Korea; soprano37@naver.com; 3 Department of Nuclear Medicine, Keimyung University Dongsan Hospital, Keimyung University School of Medicine, Daegu 42601, Republic of Korea; hwkim.nm@gmail.com; 4 Department of Nuclear Medicine, Daegu Catholic University Medical Center, Daegu Catholic University School of Medicine, Daegu 42472, Republic of Korea

**Keywords:** artificial intelligence, positron emission tomography, metabolic tumour volume, total metabolic tumour volume, prognosis, systematic review

## Abstract

**Background/Objectives**: Artificial intelligence (AI) can automate tumour-burden measurement on positron emission tomography (PET), including metabolic tumour volume (MTV) and total metabolic tumour volume (TMTV). This review evaluated prognostic evidence for AI-derived PET tumour-burden biomarkers, particularly comparisons with expert measurements. **Methods**: PubMed and Embase were searched on 29 August 2026; supplementary searching closed on 9 September. Revision follow-up used an export dated 13 October 2026 while retaining the original temporal eligibility boundary. English-language original reports evaluating AI-derived PET tumour burden and clinical outcomes were eligible. Core comparative and Contextual evidence were synthesized narratively by cancer, tracer, measurement, outcome and validation setting, accounting for shared cohorts. AI-assisted extraction and appraisal underwent human verification, without fully independent duplicate extraction or appraisal. Search coverage was incomplete, and no formal certainty-of-evidence grading was performed. The review was registered in PROSPERO (CRD420261497171). **Results**: We included 35 reports (13 Core and 22 Contextual) from 31 catalogue families; these families do not represent counts of independent validation cohorts. Selected studies reported associations between higher AI-derived MTV or TMTV and poorer outcomes, with confidence limited by confounding, threshold selection and validation design. Several reports found prognostic estimates similar to expert measurements without establishing equivalence. Heterogeneity in populations, outcomes, predictor scales and thresholds precluded pooled hazard ratios; incomplete paired uncertainty prevented pooling between-method differences. Of 62 prognostic factor assessment units, 37 had high risk of bias for confounding and 50 for statistical analysis and reporting. External validation of fixed prognostic thresholds remained limited. **Conclusions**: AI-derived MTV and TMTV showed prognostic associations in selected cancer settings, with limited confidence in their independence and transportability. Evidence does not establish interchangeability with expert measurements or improved outcomes from biomarker-guided treatment. Independent validation is needed before clinical use.

## 1. Introduction

Artificial intelligence (AI) enables automated lesion identification and segmentation on positron emission tomography/computed tomography (PET/CT), providing a means of quantifying tumour burden across multiple disease sites. The prognostic value of these measurements, however, cannot be inferred from lesion detection or segmentation performance alone. Their clinical interpretation requires evidence that automated measurements retain prognostic information and perform reliably in the intended setting. Studies in lymphoma, lung cancer and head and neck cancer have examined this relationship between automated tumour-burden measurement and patient outcomes [1,2,3].

Metabolic tumour volume (MTV) represents the volume of metabolically active tumour identified by a specified segmentation procedure, while total metabolic tumour volume (TMTV) generally sums these volumes across the lesions included in the analysis. For fluorodeoxyglucose ([^18^F]FDG) PET, total lesion glycolysis (TLG) combines lesion volume with the mean standardized uptake value, with lesion-level products summed when multiple lesions are assessed. These measures characterize tumour burden beyond the maximum standardized uptake value, which describes the highest measured uptake. Their interpretation also depends on the anatomical coverage of the measurement. Primary-tumour MTV, primary-plus-nodal burden and whole-body TMTV may share the same unit but represent different anatomical extents of disease [1,2,4].

The biological meaning of a burden measure also depends on the radiotracer. Prostate-specific membrane antigen (PSMA)-positive total tumour volume reflects PSMA-targeted uptake rather than glycolytic activity. Similarly, choline-derived prostate uptake, methionine-associated marrow activity and measures obtained with tracers targeting C-X-C chemokine receptor type 4 (CXCR4) reflect distinct biological and anatomical characteristics. We therefore use PET tumour burden as an umbrella term, while retaining MTV, TMTV, TLG or total tumour volume (TTV) according to the tracer and measurement reported in each study [5,6,7,8].

The clinical value of these measures also depends on how they are obtained. Reproducible quantification could support baseline risk assessment, longitudinal monitoring and stratification in clinical research. Expert measurement requires identification of malignant lesions, separation from physiological uptake and decisions about low-uptake or poorly delineated disease. Automation may reduce manual contouring, but these decisions remain reflected in the training labels and in the rules used to identify, segment and quantify lesions. Evaluation of an AI-derived biomarker therefore needs to consider the complete measurement procedure, including anatomical masks, uptake thresholds and post-processing, alongside the model itself [1,9,10].

Agreement with expert segmentation does not necessarily indicate comparable prognostic performance. An automated method may underestimate tumour volume while preserving patient ranking and survival discrimination. Conversely, a high segmentation overlap score may conceal measurement differences that alter risk classification near a prognostic cutoff. In the REMARC analysis by Capobianco and colleagues, automated and reference TMTV differed in their distributions, although both were associated with survival [1]. These findings support direct assessment of prognostic performance and indicate that an expert-derived cutoff requires validation before it is applied to automated measurements.

Prognostic association alone does not establish clinical usefulness. A statistically significant hazard ratio (HR) indicates an association within the fitted model, but does not show whether the biomarker improves prediction beyond established clinical predictors or provides accurate estimates of absolute risk. Nor does it establish whether using the biomarker to guide treatment improves patient outcomes. To assess comparative prognostic performance, AI-derived and expert-derived measurements should be evaluated in the same patients, for the same outcome and time horizon, using compatible predictor definitions and adjustment sets. Similar HRs or overlapping confidence intervals (CIs) alone do not establish equivalence.

We conducted this systematic review to evaluate the prognostic evidence for AI-derived PET tumour-burden biomarkers in cancer, with particular attention to comparisons with expert-derived measurements. MTV and TMTV remained the central focus; TLG and other tracer-defined volumes were interpreted separately. Projection-based surrogates, dissemination measures and comparisons involving anatomical gland measurements or clinical gross tumour volumes were distinguished from directly comparable volumetric burden measures. We examined whether these comparisons used corresponding patient populations and compatible measurement definitions, and whether the uncertainty in differences between methods could be assessed. Secondary aims were to describe applications across cancer types and radiotracers, distinguish segmentation validation from prognostic validation, and identify requirements for clinical translation. Studies without a direct comparison with expert-derived measurements were considered separately to provide clinical and methodological context.

## 2. Materials and Methods

### 2.1. Review Framework

This systematic review was registered in PROSPERO on 6 September 2026 (CRD420261497171). Title and abstract screening began on 2 September 2026, before registration, but data extraction had not started at the time of registration. Reporting followed PRISMA 2020 and PRISMA-S [11,12]. The review examined the prognostic performance of PET tumour-burden measures generated using AI-assisted or automated procedures in patients with cancer, with particular emphasis on comparisons with expert-derived measurements. For assessing independent evidence, we considered the combination of cohort, outcome and assessment timepoint. Publications reporting distinct analyses of a shared patient source were retained as separate reports, while overlap between their patient populations was considered during synthesis. Registered methods: https://www.crd.york.ac.uk/PROSPERO/view/CRD420261497171 (accessed on 6 September 2026). A full protocol was prepared as an internal working document but has not been publicly released. Differences between the planned and implemented methods are described in Supplementary Methods S6.

### 2.2. Eligibility and Evidence Tiers

English-language original reports were eligible if they evaluated an AI-enabled procedure that generated a PET-derived tumour-burden measure and reported a relevant clinical outcome analysis. Non-English reports were excluded during screening because of limited translation resources. Eligible procedures included direct lesion segmentation and AI-generated anatomical masks combined with PET quantification. PET-free surrogates, AI prognostic models that did not generate an eligible PET burden measure, and segmentation studies without a relevant clinical outcome analysis were outside the review scope. Composite predictors incorporating tumour burden were identified separately to avoid attributing their prognostic performance to the burden measure alone.

Core/Contextual criteria were documented on 29 August 2026, before formal screening began. Core criteria required AI-derived PET tumour burden, expert-derived measurements in the same cohort and results permitting direct comparison of prognostic performance; the linked framework also allowed classification agreement. Otherwise eligible prognostic reports without the required comparative evidence were classified as Contextual. Kendrick was retained as a boundary Core report for same-patient response classification, while its unequal-sample survival estimates were excluded from aligned prognostic comparisons. Core reports provided the principal comparative evidence, whereas Contextual reports informed clinical applications, prognostic associations and methodological limitations. These categories described the role of each report in the synthesis and did not represent risk-of-bias ratings. A Core classification did not imply that every result within a report was directly comparable or suitable for pooling. Comparator availability, correspondence between analyzed patient populations and compatibility of measurement definitions were therefore assessed for each analysis. During revision, a report-level evidence map linked all 35 classifications to these criteria and documented boundary cases (Supplementary Methods S20). Anatomical whole-gland PET measurements were retained as Contextual evidence even when AI and manual prognostic results were available, because they did not isolate lesion-defined tumour burden [13]. This construct boundary was clarified during revision and was not a fully prespecified operational rule.

### 2.3. Information Sources and Searches

PubMed and Embase were searched on 29 August 2026, with supplementary backward and forward citation searching and Google Scholar screening. The database strategies combined terms for PET, AI, segmentation or tumour burden, and malignancy. Prognostic terms were not mandatory because relevant outcome analyses could be secondary components of segmentation studies. Under a prespecified conditional rule, conference publication types were excluded from the Embase search when they accounted for more than 30% of the unrestricted results. Trial-registry records were retained separately and excluded from the bibliographic export. Supplementary searching was documented in a closure record finalized on 9 September 2026. The executed database strategies, search accounting and available supplementary-search records are provided in Supplementary Methods [12]. Temporal eligibility was determined by the first online publication date. Revision follow-up used a search export dated 13 October 2026, with dispositions finalized on or before 15 October. Reassessment retained 9 September 2026 as the temporal eligibility boundary; this was not a comprehensive update through the revision date. Web of Science, Scopus, and IEEE Xplore were not searched because institutional subscription access was unavailable when the review was initiated.

Backward citation searching covered the reference lists of the original 31 included reports. Forward citation searching used PubMed cited-by links for 10 seed reports on 29 August 2026. The first 200 ranked Google Scholar results were also screened. Reference-list citations were counted separately from unique database records, and previously identified reports were not counted as newly discovered studies. Reports already present in the database results whose eligibility was reassessed were distinguished from eligible reports newly identified through supplementary searching. Byungwook Choi and Sang Jun Byun independently screened the titles and abstracts of all 3188 records and recorded their decisions separately; disagreements were reconciled before full-text assessment. Hae Won Kim, a nuclear medicine physician, verified the full-text eligibility decisions before submission, and Sang Jun Byun checked all Core reports. Questions raised during verification were checked and discussed with Byungwook Choi, who made the final decisions.

The revision search export dated 13 October 2026 contained 2402 retained records. After linking 18 duplicates, 2384 active records remained: 2092 screening exclusions and 292 follow-up records (291 exclusions and one Contextual inclusion, Küper et al.). All record-level dispositions were adopted by the author on or before 15 October 2026. The five duplicates within the 297 raw follow-up rows are included in the 18 links and are not subtracted twice. Thus, the revision branch comprises 2383 exclusions and one inclusion. These are AI-assisted, author-adopted decisions, not independent duplicate screening; source access ranged from abstracts to complete reports. Revision exclusions are not added to the original 69 full-text exclusions. MAA-Net was assessed from its abstract and selected main-text excerpts, without its complete text. Following AI-assisted review, Sang Jun Byun independently reviewed the additional and changed revision materials, and Byungwook Choi made the final decisions. This verification did not constitute independent duplicate extraction or appraisal from scratch, or independent duplicate screening of all 2384 revision records. The absence of independent duplicate extraction and appraisal remains a methodological limitation.

### 2.4. Extraction and Measurement Definitions

Data extraction linked each report to its study, cohort family, patient cohort, model, burden measure, outcome and effect estimate. Extracted fields included cancer type and treatment setting, radiotracer, patient and scan counts, model architecture, data splitting, reference measurement procedures, manual correction, burden definition, anatomical coverage, predictor transformation, outcome definition, follow-up, effect estimates and uncertainty. Sample sizes were recorded for each analysis when they differed from those of the segmentation dataset or parent cohort. AI assisted with data extraction, reconciliation and language editing. Hae Won Kim verified the extracted data against the original reports before submission. Questions were checked and discussed with Byungwook Choi, who finalized the extraction. We sought to extract eligible prognostic results as comprehensively as possible from the available reports. Study investigators were not contacted to request additional data. Source locations were recorded for numerical estimates. Discrepancies between abstracts, main texts and tables were documented for clarification rather than averaged or silently resolved.

Baseline burden, longitudinal change and composite response criteria were treated separately. Absolute and relative changes were not combined, and volumetric measures, uptake-weighted volumes and two-dimensional projection surrogates were distinguished. We also separated external testing of a segmentation model from external validation of a prognostic threshold or prediction model. A new imaging cohort was not considered an independent prognostic validation cohort if its outcomes were used to select the threshold subsequently evaluated in the same patients.

### 2.5. Critical Appraisal

Prognostic factor associations were assessed using the six Quality in Prognosis Studies (QUIPS) domains, with separate assessments for each outcome and cohort [14]. Segmentation agreement was evaluated using an eight-domain form developed for this review, termed Form S, which is not a validated instrument. AI-assisted assessment and consistency checking covered 62 prognostic factor assessment units and six segmentation evaluation scopes. Hae Won Kim verified the original QUIPS and Form S assessments before submission, and Byungwook Choi finalized the judgments after discussion of questions. During revision, Choi finalized two additional QUIPS units for Liang et al. after AI-assisted review of the main article and Supplementary Information. On 15 October 2026, Choi adopted two outcome-specific assessments for Gutsche et al. and four factor/outcome assessments for Küper et al. after reviewing the AI-assisted proposals and supporting rationales. These six assessments concern two cohorts and do not represent independent replications.

Clinical prediction models were assessed separately using a framework based on PROBAST + AI, distinguishing development quality, risk of bias in model evaluation and applicability [15]. Hae Won Kim verified the model assessments before submission; Byungwook Choi finalized 119 model-assessment records and 476 domain judgments. These comprised 39 development and 80 evaluation records across ten reports, not independent models or cohorts. Full-text methods were available for nine reports, whereas one proceedings report was assessed from its abstract. During revision, applicability explanations were adopted for all 119 records, covering participants, predictors and outcomes in relation to the intended prediction time. The researcher subsequently adopted 198 item-response changes and 45 retain-response recommendations; 22 analysis-domain and overall evaluation judgments changed from Unclear to High. Four Kovacs development assessments and the review-level PICOTS clarification were adopted, with 357 applicability explanations. The complete official form was checked for structural correspondence, but source-level validity of every response has not been verified. The earlier review-specific assessment grid was retained for documentation and was not treated as a validated-tool assessment.

Human verification and final adjudication did not constitute fully independent duplicate appraisal. No numerical quality score or formal certainty-of-evidence rating was assigned. CLAIM 2024 and TRIPOD + AI informed the assessment of reporting considerations [16,17]. Assessment scope, domain judgments and limitations are provided in Supplementary Appraisal.

### 2.6. Comparative Synthesis

Evidence was synthesized by cancer type, radiotracer, clinical setting, outcome and measurement definition. Comparisons between AI-derived and expert-derived results considered the analyzed patient populations, predictor scale, outcome horizon and adjustment variables. HRs for continuous transformed predictors were not directly compared with those for dichotomized predictors. Analyses using separately optimized thresholds for each measurement method were treated as different risk contrasts, even when they involved the same patients. Univariable associations were distinguished from associations adjusted for clinical covariates. Adjustment reported for a separate model was not attributed to an extracted univariable estimate. HRs with unverified predictor coding or transformation were described but excluded from quantitative synthesis.

For estimates derived from the same patients, uncertainty in the difference between methods was not calculated under an assumption of independence. Correlations between tumour-burden measurements were not used as substitutes for covariance between prognostic effect estimates. Published paired comparisons were interpreted according to their reported methods, and study-specific non-inferiority margins were not adopted as review-wide criteria. Quantitative pooling required at least two independent cohort contributions with compatible clinical settings, corresponding prognostic contrasts and sufficient information to estimate uncertainty. When these conditions were not met, findings were synthesized narratively using an approach informed by SWiM [18]. No overall prognostic HR was pooled across radiotracers, cancer types or burden definitions. Qualitative confidence was interpreted by disease and outcome in relation to the amount and independence of evidence, measurement and comparator directness, consistency, precision, confounding and analysis risk, and validation setting. No formal certainty grades were assigned.

During revision, we performed a descriptive sensitivity synthesis restricted to Core reports and then to aligned comparison records. Multivariable-model AUC comparisons were considered separately from burden-factor evidence. These restrictions did not treat repeated analyses as independent studies and did not constitute a pooled-effect sensitivity analysis.

## 3. Results

### 3.1. Study Selection and Evidence Structure

**Figure 1:**
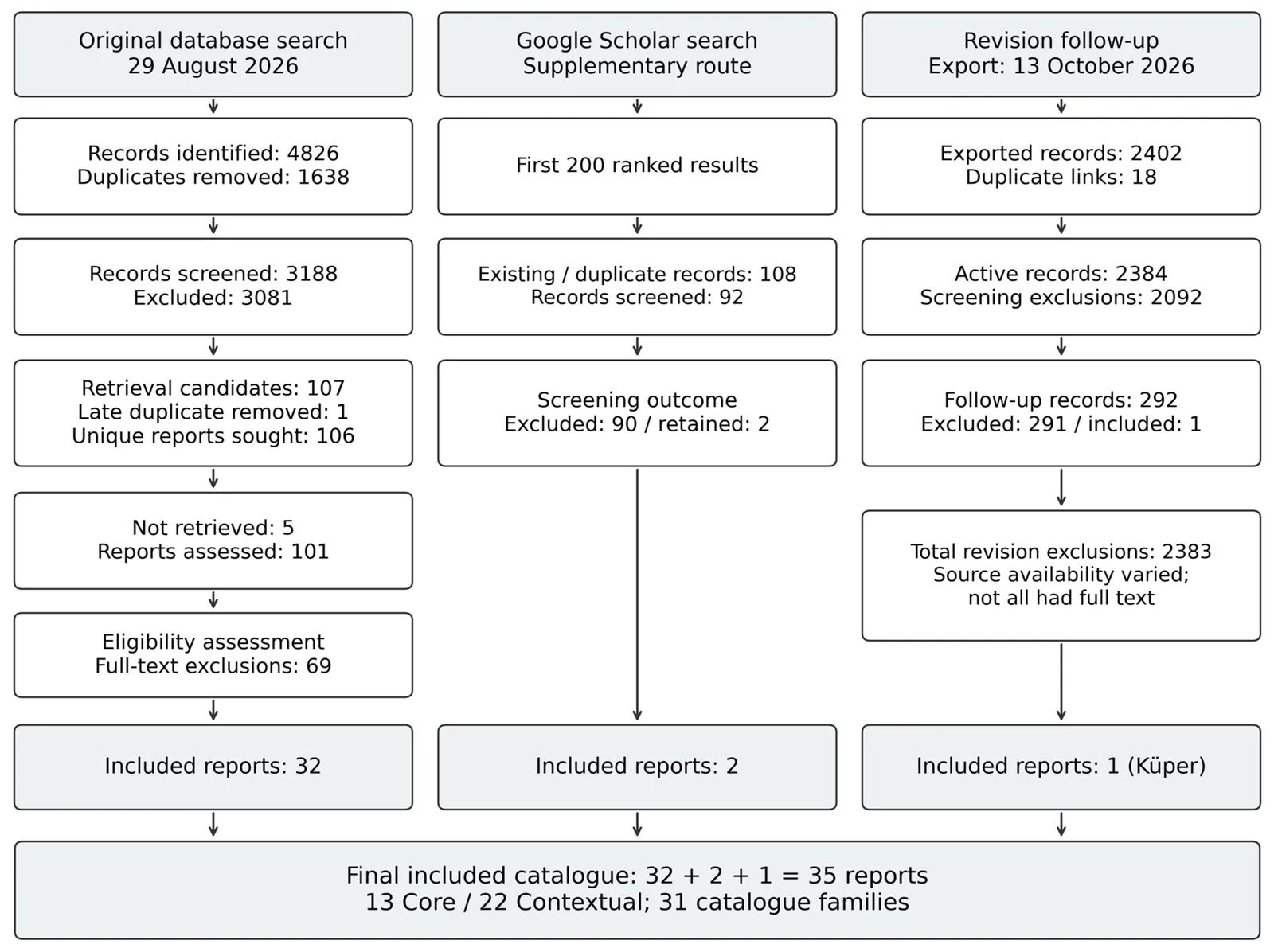
Study-selection accounting by search route (32 + 2 + 1 = 35 included reports). Forward citation searching identified 159 unique records: 57 already identified and 102 screened and excluded. Backward searching covered 988 citation instances without additional included reports. Revision exclusions include assessments without full text and remain separate from original full-text exclusions. Incomplete Google Scholar records limit cross-route matching (Supplementary Methods S24).

The original database searches identified 4826 records, comprising 1786 from PubMed and 3040 from Embase. After removal of 1638 duplicates, 3188 records underwent screening. In the reconciled original database branch, 3081 records were excluded and 107 records were considered for full-text retrieval. Removal of one additional duplicate left 106 unique reports sought for retrieval. Five reports could not be retrieved. Of the 101 reports assessed in this branch, 69 were excluded and 32 were included. Two additional Contextual reports were identified through Google Scholar. After re-inclusion of Le Goff et al. from the original title/abstract exclusions, Küper et al. were adopted as Contextual evidence from the separate revision-stage follow-up set, bringing the current catalogue to 35 reports from 31 catalogue families, comprising 13 Core and 22 Contextual reports. Figure 1 and Supplementary Methods S24 present the original and revision branches separately, preserving their different assessment levels. Liang et al. were included on reassessment of the original database results. Gutsche et al. were transferred from the previous E2 exclusion category to Core inclusion; that reclassification did not add an assessed report. Wang et al. remained Contextual after correction of the comparator attribution [19,20].

Backward citation searching examined 988 references cited across the original 31 included reports. This count represented citation instances rather than unique records. No additional eligible report absent from the database results was identified. One report already retrieved through the database searches was reassessed and included after its AI-generated burden measure and clinical outcome analysis were verified. This reassessment was recorded separately from the identification of new reports. Forward citation searching identified 159 unique records, including 57 already present in the database results. The remaining 102 records were independently screened and excluded, yielding no additional eligible report.

Additional source checking of ten reports retained in the separate revision-stage follow-up set supported nine E1 exclusions, adopted by the researcher on 15 October 2026, because the reported PET measurement procedures had no learning-based component, and one E2 exclusion because the AI-derived burden itself was not evaluated prognostically. The researcher also adopted the E2 exclusion. These ten decisions belong to the revision-stage follow-up branch and are included in its 291 exclusions. Küper et al. were adopted as Contextual evidence on researcher reassessment; four QUIPS scopes were finalized on 15 October 2026 (Supplementary Methods S12) [20].

The clinical distribution of included reports is summarized in Table 1, and all 35 reports are listed in Supplementary Data. Comparative evidence was concentrated in [^18^F]FDG imaging of lymphoma and head and neck cancer. Other Core reports evaluated lung cancer, thymic epithelial tumours, melanoma, breast cancer, PSMA imaging in prostate cancer and FET imaging in glioma. Contextual reports broadened the evidence to include myeloma and settings involving chimeric antigen receptor T-cell (CAR-T) treatment, immunotherapy, taxane chemotherapy and radiopharmaceutical therapy. The extraction dataset contained 37 model records, 161 burden-related records, 54 outcome records and 361 effect records. These counts describe the structure of the extracted data and do not represent independent models, patient populations or replications of an effect.

The newly assigned CF031 is a provisional catalogue family; patient-level independence and overlap with segmentation-development data have not been established. Two Le Goff factor scopes were finalized by the researcher on 15 October 2026.

### 3.2. Lymphoma

Lymphoma studies showed that automated tumour-burden measurements can retain prognostic information despite substantial differences from expert-derived measurements. Among 280 analyzable patients in the REMARC cohort, median automated and reference TMTV values were 110 and 240 cm^3^, respectively. For four-year progression-free survival (PFS), automated TMTV yielded an area under the receiver operating characteristic curve (AUC) of 0.63 and an HR of 2.3 (95% CI 1.5–3.6), compared with an AUC of 0.69 and an HR of 2.6 (95% CI 1.6–4.1) for reference TMTV. The HRs were based on method-specific cutoffs of 171 and 242 cm^3^, respectively. These findings support the prognostic relevance of automated TMTV, but the similar HRs do not establish interchangeable measurements or comparable performance at a common threshold [1].

Projection-based assessment offered a different approach to quantifying lymphoma burden. A method using PET maximum-intensity projections to derive two-dimensional measures of tumour burden and dissemination was developed in REMARC and evaluated in the independent LNH073B cohort, with outcome analyses involving 287 and 95 patients, respectively. The burden measure represented an area in cm^2^ rather than a volume in cm^3^ and was therefore considered a surrogate rather than a direct volumetric TMTV measurement. Its prognostic performance supported the potential use of projection-based measures but did not establish their interchangeability with volumetric TMTV. Because the REMARC population overlapped with the preceding volumetric analysis, this contribution was not treated as independent replication [1,23]. The predictor coding or transformation underlying 16 extracted HRs could not be verified. These estimates were excluded from quantitative synthesis, while the reported AUCs were retained for descriptive comparison [23].

The PETAL analysis evaluated PFS in 534 patients from the 716-patient population used for segmentation development. Applying the same threshold of 210.2 mL, optimized using manual TMTV, yielded HRs of 3.7 for convolutional neural network (CNN)-derived TMTV and 3.5 for manual TMTV, although CIs were not available in the extracted results. At this threshold, risk classification differed between the two methods in 3.6% of patients. These findings suggest similar risk classification at a common threshold, although the threshold was selected using outcome data and no paired test of the difference between HRs was reported. External segmentation testing on a separate benchmark did not establish external validation of the prognostic findings [10]. Supplementary segmentation analyses included 485 PET-negative scans among 1192 scans, but the available description did not establish whether all evaluated predictions were generated independently of model training [10]. Confidence in the lymphoma comparisons was limited by different effect measures and threshold strategies, overlapping REMARC analyses and incomplete comparative uncertainty; these findings did not establish equivalent prognostic performance [1,10,23].

Le Goff et al. provided additional Contextual evidence in aggressive large B-cell lymphoma. Among 102 patients with PET measurements, baseline TMTV > 200 mL had a crude PFS HR of 4.58 (95% CI 1.59–13.15) and an adjusted HR of 2.23 (0.70–7.12). The outcome-derived threshold and absence of a paired expert-burden prognostic comparison limit interpretation [26].

Multicentre diffuse large B-cell lymphoma (DLBCL) cohorts also provided evidence for deep-learning PET biomarkers as part of a broader prognostic-index programme. In an external R-CHOP cohort of 194 patients, the two-year PFS AUC was 0.61 for AI-derived TMTV and 0.59 for AI-derived TLG. Both estimates concerned automated measurements and therefore did not provide a prognostic comparison with expert-derived tumour burden. These results were considered Contextual evidence and were distinguished from the performance of the combined prognostic model incorporating molecular and clinical information. Any benefit of that model could not be attributed to automated TMTV alone [21].

Additional studies supported the prognostic relevance of automated PET measurements across different lymphoma populations. In 1268 patients with previously untreated DLBCL from the GOYA trial, a model combining automated imaging measurements with clinical variables was developed in 846 patients and evaluated in a prespecified holdout set of 422 patients. The combined model produced greater separation of PFS and overall survival (OS) risk groups than the clinical-only model, supporting the potential contribution of imaging information to risk stratification, although this benefit could not be attributed to TMTV alone [9]. In another DLBCL study, a segmentation model developed in 297 patients was evaluated in an external cohort of 117 patients, in which higher automated TMTV was associated with shorter PFS and OS. These findings supported its prognostic relevance, although the report did not establish whether the prognostic cutoff had been fixed before outcome analysis in the validation cohort [24].

The association between higher tumour burden and poorer outcomes was also observed in 37 patients with aggressive B-cell lymphoma receiving CAR-T therapy. Higher baseline AI-guided TMTV and elevated lactate dehydrogenase were independently associated with shorter PFS, suggesting a potential role in pretreatment risk stratification that requires confirmation in larger cohorts [22]. In a separate study of 82 patients with stage I–II follicular lymphoma treated with radiotherapy, PET features derived from the consensus or union of AI-generated masks yielded the best reported cross-validated C-indices of 0.64 and 0.65 for progression prediction, compared with 0.56 for consensus masks combining threshold-based and AI-based segmentation. These results suggested that the choice of segmentation aggregation method could influence prognostic performance, but discrimination remained modest, and the abstract-level report provided neither external validation nor a prognostic comparison with expert-derived tumour burden [25].

### 3.3. Head and Neck Cancer

Head and neck cancer studies evaluated whole-disease tumour burden, primary-plus-nodal TLG, radiotherapy-planning volumes and longitudinal changes. In a study of patients with head and neck squamous cell carcinoma, OS analyses included 585 patients in the main cohort and 142 patients in an external cohort. Higher total tumour burden was associated with shorter OS using either manual or CNN-derived measurements. When a common threshold based on the median manual measurement in the main cohort was applied to the external cohort, the HRs were 1.8 for manual measurements and 1.9 for CNN-derived measurements. These findings supported the prognostic relevance of automated tumour-burden assessment in an external population, but the similar HRs did not establish equivalent prognostic performance because uncertainty in their paired difference was not assessed [3].

Beyond volumetric measures, a study of 366 patients with oropharyngeal carcinoma treated with curative radiotherapy or chemoradiotherapy evaluated combined TLG of the primary tumour and involved lymph nodes and found that higher values were associated with a greater risk of distant metastasis [4]. A TLG threshold of 290, selected in this exploratory cohort, was subsequently applied to an external validation cohort of 65 patients. Above this threshold, the HRs were 3.1 for CNN-derived TLG and 3.47 for manually derived TLG in the same validation patients. These findings supported the ability of automated TLG to identify patients at higher risk of distant metastasis, although the univariable analyses did not establish independence from clinical confounders. CIs for the validation HRs were unavailable, and inconsistent reporting of TLG units limited interpretation of the threshold [4]. Both cohorts overlapped with the datasets used in the preceding head and neck cancer study and were therefore not considered independent replication across reports [3,4]. Thus, external outcome evaluation provided relevant support, but shared source patients, different outcomes and burden definitions, and unadjusted analyses limited confidence in independent replication of the AI-versus-expert contrast [3,4].

In 540 patients with head and neck cancer, AI-derived PET-avid tumour volume was compared with manually delineated clinical gross tumour volume for recurrence prediction. The authors reported that AUC and Brier scores met their non-inferiority criteria at one and three years, but not at five years [27]. The supplementary code separately refitted both models and evaluated them in the same dataset. The supplied non-inferiority calculation derived its standard error from the reference-model confidence interval without explicitly estimating uncertainty in the paired difference. These results therefore require cautious interpretation and do not establish fixed-coefficient external prognostic validation. The manual volumes included non-FDG-avid components, whereas the automated volumes represented PET-avid disease.

Contextual studies examined both longitudinal volume changes and baseline MTV. In an external cohort of 50 patients with head and neck cancer, greater reduction in AI-derived PET-avid tumour volume after radiotherapy or chemoradiotherapy was associated with better locoregional control by log-rank analysis. However, the Cox estimate remained uncertain (HR 0.13, 95% CI 0.02–1.04), with only nine locoregional failures. The segmentation model was applied without retraining, but the prognostic threshold was based on the validation cohort median [28]. In 83 patients with non-metastatic nasopharyngeal carcinoma, higher AI-assisted primary-tumour MTV was associated with shorter PFS and OS after adjustment for age, although its three-year AUCs were 0.60 and 0.71, respectively. These findings supported a prognostic association but showed limited discrimination, particularly for PFS [29]. In another cohort of 74 patients with head and neck cancer, higher automated MTV was associated with shorter OS in univariable analysis, but the association was not retained in the reported multivariable model [30]. None of these studies provided a direct prognostic comparison between automated and expert-derived volume measurements [28,29,30].

### 3.4. Other [^18^F]FDG-Avid Solid Tumours

In a held-out test cohort of 106 patients investigated for suspected lung cancer, higher TLG was associated with shorter OS using either automated or manual measurements. The HR per unit increase in log10(TLG + 1) was 1.64 (95% CI 1.21–2.21) for CNN-derived TLG and 1.54 (95% CI 1.14–2.07) for manually derived TLG. These findings supported the preservation of prognostic information in an uptake-weighted burden measure, although the cohort also included lung metastases, lymphoma and benign or unresolved diagnoses, limiting interpretation specifically for primary lung cancer. Both methods were evaluated in the same patients using the same predictor transformation, but uncertainty in the paired difference was not reported. The test cohort was also not fully external because its institution had contributed to model training [2]. Because these associations were univariable, they did not establish independence from diagnosis or other clinical confounders [2].

In a feasibility study of patients with non-small cell lung cancer, prognostic models were developed in 150 patients and evaluated in a separate cohort of 89 patients scanned at the same imaging centre. Automated segmentation provided MTV, TLG and other PET features for predicting death within one year. Across six models, AUCs in the complete validation cohort ranged from 0.58 to 0.68, indicating limited discrimination. Higher AUCs in exploratory stage-balanced subsamples did not establish improved performance in the full cohort, which contained only eight deaths within one year. These results highlighted the dependence of prognostic performance on cohort composition and did not establish the superiority of more complex models or equivalence between automated and expert-derived MTV [31].

In 114 patients with surgically resected thymic epithelial tumours, higher automated and manual MTV were associated with a greater risk of recurrence. The HRs per unit increase in log10-transformed MTV were 2.11 (95% CI 1.09–4.06) for automated measurements and 2.43 (95% CI 1.33–4.45) for manual measurements. TLG showed similar associations with recurrence, whereas the univariable OS estimates were imprecise; their confidence intervals included 1, and only eight deaths were observed. These findings supported the prognostic relevance of automated MTV for recurrence, but did not establish an association with OS [32]. In a separate cohort of 50 patients with predominantly advanced breast cancer, a model trained on lymphoma and lung cancer was applied without breast-cancer-specific training. Higher automated and manual whole-body MTV were both associated with shorter OS, with concordance estimates of 0.65 and 0.69, respectively. These results suggested that automated MTV could retain prognostic information when applied to another cancer type, although the small cohort limited generalizability [33]. The nonsignificant concordance comparison did not establish equivalence, and the organ-specific HRs arose from separate stepwise models rather than a common fixed adjustment set [33].

In non-metastatic breast cancer, Liang et al. evaluated MTV and TLG derived from automated segmentation of the primary tumour and FDG-avid lymph nodes. Their single-centre cohort included 1210 patients, with internal test analyses of 610 patients for OS and 570 for disease-free survival (DFS). High versus low MTV was associated with OS (HR 2.12, 95% CI 1.28–3.51) and DFS (HR 1.73, 95% CI 1.15–2.61). TLG was associated with OS, whereas its DFS confidence interval included the null. These unadjusted burden-specific results were retained as Contextual evidence and distinguished from the multimodal MOPS model. Prognostic performance was not compared with manually derived burden measurements [34].

For advanced melanoma, a comparative study examined whether automated baseline TMTV retained prognostic information in patients receiving pembrolizumab. Among 69 patients with unresectable stage III or IV disease, higher TMTV was associated with shorter OS using either automated or manual measurements. At a common threshold of 90 mL, the HRs were 14.3 (95% CI 5.72–35.8) for automated TMTV and 12.2 (95% CI 4.95–29.8) for manual TMTV. These findings supported the prognostic relevance of automated TMTV, although the threshold had been optimized using manual measurements and outcomes in the same development cohort. A model combining automated TMTV with lactate dehydrogenase and brain metastasis status achieved AUCs of 0.76 and 0.74 for one- and two-year OS in a separate 31-patient cohort from the same institution. This validation concerned the combined model and did not independently confirm the TMTV-specific associations or establish equivalence with manual measurements [37]. In the 69-patient development cohort, four comparisons of multivariable-model AUCs used leave-one-out cross-validation and showed no detected DeLong difference. Two-year PFS AUCs of 0.42 and 0.40 for automated and manual models illustrated that numerical similarity could coexist with poor discrimination; these comparisons did not establish equivalence or externally validated standalone TMTV performance [37].

Serial PET assessment extended the melanoma evidence beyond baseline risk stratification. In a cohort of 43 patients receiving immune checkpoint inhibitors, higher AI-assisted TMTV after two and four treatment cycles was associated with shorter OS in univariable analyses, whereas baseline TMTV was not. After adjustment for clinical factors, the association remained significant only at the four-cycle assessment, suggesting that residual tumour burden during treatment may provide prognostic information [35]. A smaller study of 15 patients with metastatic uveal melanoma receiving tebentafusp found that higher TMTV at both baseline and three months was associated with shorter OS. TLG, assessed separately, showed similar associations, but the analyses relied on median-based groups and log-rank tests without reported HRs [36]. These studies supported further evaluation of serial tumour-burden measurements, although their small cohorts and absence of a manual-burden prognostic comparator limited conclusions about comparative performance [35,36].

### 3.5. Prostate Cancer, Myeloma and Glioma

PSMA PET studies extended volumetric assessment to prostate cancer, using PSMA-positive total tumour volume rather than [^18^F]FDG-derived MTV. In biochemically recurrent prostate cancer, an increase in TTV of at least 20% was associated with shorter OS using both manual and AI-derived measurements. The HRs were 2.50 (95% CI 1.35–4.63) in the 199-patient manual cohort and 3.65 (95% CI 1.23–10.89) in its nested 74-patient AI test subset. Progression according to Response Evaluation Criteria in PSMA PET/CT was also associated with shorter OS, with corresponding HRs of 3.78 and 3.75. These findings supported the prognostic relevance of automated volume assessment, but the different analysis populations prevented a same-patient comparison. These response criteria additionally incorporate new-lesion status, so their prognostic association could not be attributed to TTV change alone [39]. An earlier report from the same registered cohort family also found an association between automated baseline tumour volume and OS in 72 patients; this was retained as Contextual evidence without counting it as independent replication [40]. The same-patient response-classification comparison was considered separately from the unequal-sample survival estimates and did not establish prognostic equivalence [39].

The prognostic relevance of PSMA-positive tumour volume was also examined during systemic treatment for advanced prostate cancer. Among 107 patients with metastatic castration-resistant disease receiving lutetium-177 PSMA therapy, higher automated baseline tumour volume was associated with shorter OS, with an HR of 1.26 (95% CI 1.08–1.46) per doubling of volume. This association was derived from a single-centre cohort; a separate segmentation evaluation did not provide external prognostic validation [5]. A multicentre study of 128 patients receiving taxane chemotherapy similarly found that higher baseline PSMA-positive tumour volume and a smaller reduction during treatment were associated with shorter OS. These findings supported the potential value of both baseline burden and serial volume assessment, although the prognostic cutoffs were selected using outcomes in the same cohort [38]. Neither study provided a direct prognostic comparison with expert-derived volume measurements, and their treatment settings differed from biochemical recurrence [5,38].

Choline PET studies focused on uptake-defined burden within the prostate gland rather than whole-body tumour volume. In 43 patients with hormone-naive prostate cancer and bone metastases, larger intraprostatic abnormal-uptake volumes were associated with shorter OS using both AI-derived measurements and measurements from two expert readers [13]. Application of the related algorithm to an independent cohort of 285 patients with newly diagnosed high-risk prostate cancer showed that larger uptake-defined volumes were associated with shorter prostate cancer-specific survival after adjustment for treatment type. The association was observed in the palliative-treatment subgroup but was not demonstrated in the curative-treatment subgroup [6]. These findings supported the prognostic relevance of automated intraprostatic burden assessment, but did not establish equivalence to whole-body TMTV. Although the studies shared an algorithm-development lineage, their outcome cohorts were independent [6,13].

In multiple myeloma, AI-assisted quantification addressed metabolically active disease within the bone marrow as well as focal lesions. The CASSIOPEIA ancillary analysis included 195 patients with newly diagnosed disease and found that higher medullary TMTV was associated with shorter PFS and OS after adjustment for clinical factors, whereas focal-lesion TMTV did not show significant associations with either outcome. These findings suggested that the anatomical scope of volume measurement influenced its prognostic relevance [41]. A separate study of 44 newly diagnosed patients combined AI-generated skeletal masks with PET uptake thresholds to quantify bone marrow MTV. Higher MTV was associated with shorter PFS across several threshold definitions, including adjusted analyses, while the OS associations were less consistent. Interpretation remained exploratory because multiple thresholds were evaluated without correction for multiple testing [42]. Both studies supported further evaluation of marrow-based volumetric measures, but neither directly compared their prognostic performance with expert-derived measurements [41,42].

Other tracers provided additional perspectives on myeloma burden. In a prospective study of newly diagnosed multiple myeloma, 37 patients contributed to PFS analysis after paired [^11^C]methionine and [^18^F]FDG PET/CT. Higher AI-assisted methionine-derived bone marrow MTV was associated with shorter PFS after adjustment for beta-2 microglobulin and bone marrow plasma-cell percentage. The reported AUCs were 0.743 for methionine-derived MTV and 0.719 for [^18^F]FDG-derived MTV, but this numerical difference did not establish superiority, and the thresholds were selected within the same small cohort [7]. In a different treatment setting, 22 patients with relapsed or refractory myeloma contributed 24 cycles of CXCR4-directed radiopharmaceutical therapy. Greater CXCR4-positive volume and higher [^18^F]FDG-derived TLG in medullary lesions positive on both scans were associated with shorter OS. Interpretation was limited by uncertainty about patient-versus-cycle analysis and the use of approximate event-rate ratios rather than Cox-derived HRs [8]. These exploratory findings concerned tracer-specific marrow and lesion measures and should not be interpreted as validation of [^18^F]FDG whole-body TMTV.

Küper et al. evaluated 73 patients with prostate cancer who underwent paired PSMA PET examinations without LuPSMA therapy. Baseline body-weight-adjusted PSMAvol was associated with OS (HR 3.920, 95% CI 2.127–7.224); the change estimate was 6.502 (1.018–41.552) after adjustment for PPP and RECIP. These are continuous body-weight-adjusted effects, not per-mL estimates. The adopted segmentation method links neural-network anatomical masks with PET quantification, although implementation of the learning component is inferred from the cited method. No paired expert prognostic comparison was reported. Clinically selected repeat imaging, heterogeneous treatment and uncertain survival time origin limit interpretation [20].

In a small retrospective glioma response cohort, automated and physician-assessed 18F-FET PET MTV changes both showed OS separation, whereas PFS separation was not statistically significant (network, *p* = 0.39; physician, *p* = 0.12). The report described 33 patients, but the initial risk sets in Figure 5 of that report totaled 32. These unadjusted findings do not establish independent prognostic value or interchangeability. Independence of the response cohort from segmentation development and the handling of survival time relative to post-treatment measurement were not established [19].

### 3.6. Validation and Limitations of Quantitative Synthesis

External validation addressed different components of the measurement and prognostic workflow. Of the 36 model records, 18 included some form of external evaluation, but these did not represent 18 externally validated prognostic models. Evaluations ranged from segmentation testing in independent imaging datasets and application of pretrained models to another cancer type to testing a fixed prognostic model in an independent cohort. External segmentation performance alone therefore did not establish that an MTV or TMTV threshold retained its prognostic value in a new population. The measurement procedures also differed in whether automated contours were used directly, reviewed without modification, or manually corrected before quantification.

The 13 Core reports provided several types of comparative evidence (Table 2). Of the 69 previously documented comparison links, 23 concerned tumour-burden associations or discrimination measures evaluated in the same patients using compatible definitions. Other links involved different contouring targets, method-specific thresholds, unequal nested samples, projection-based surrogates, or comparisons across tracers and measurement types (Table 3). One link initially considered an AI-versus-expert comparison was found to compare two AI-derived biomarkers and was excluded from the comparative synthesis. These links represented individual documented comparisons rather than independent studies or estimates necessarily suitable for pooling. Supplementary Methods S9, Tables S1 and S2, present all 23 aligned records, including 16 HR comparisons, six AUC comparisons and one concordance comparison. Four AUC records evaluate multivariable models rather than standalone tumour burden. Two additional qualitative OS/PFS comparisons from Gutsche et al. are recorded separately; they report log-rank results without HRs or paired performance-difference estimates and do not enlarge the 23-record aligned set [19]. Four Jemaa same-patient comparisons are recorded separately as method-specific or unresolved-threshold comparisons; they do not enlarge the aligned set [9].

Even among clinically relevant comparisons, the available data were insufficient to pool differences in prognostic performance between automated and expert-derived measurements. Some studies reported formal comparative tests, whereas others provided only separate estimates for each method. Compatible independent cohorts with sufficient information to quantify uncertainty in the paired difference were lacking. Consequently, the evidence supported a narrative assessment of comparative performance but neither established general equivalence nor demonstrated a consistent difference between methods. In lymphoma, the aligned records use different effect measures and threshold strategies; the aligned head and neck reports share source patients and differ in burden definition and outcome. Each remaining aligned disease group contains one report. Cohort dependence, predictor transformations, thresholds, adjustment, outcome horizons and available comparative uncertainty are detailed in Supplementary Methods S9.

The descriptive Core-only sensitivity synthesis retained 13 of the current 35 reports. The 23 aligned comparisons arose from eight reports and comprised 16 HR comparisons, six AUC comparisons and one concordance comparison. Four AUC comparisons evaluated multivariable models; separating these left 19 comparison records, which were not all univariable analyses. Across these restrictions, evidence of prognostic association remained in several settings, but general equivalence or superiority of AI-derived measurements over expert measurements was not established. Null and imprecise results were retained. Heterogeneity, cohort overlap and incomplete paired-difference uncertainty continued to preclude a pooled comparative estimate (Supplementary Methods S18). This restriction did not constitute a risk-of-bias-exclusion or external-validation-only sensitivity analysis. Supplementary Table S2a consolidates cohort-dependence and validation qualifications for the aligned reports and the separate Kovacs comparison. The aligned set is not an external-prognostic-validation-only subset.

Model-specific assessments identified both documented methodological problems and uncertainties arising from incomplete reporting. In the CXCR4-directed therapy study, two treatment cycles with insufficient follow-up were classified as non-response [8]. In the melanoma study evaluating automated TMTV, patients with unknown survival status were excluded from performance evaluation [37]. These procedures supported high-risk judgments for the affected evaluations. By contrast, the chronology of predictor selection in the multicentre DLBCL study was insufficiently reported to determine whether information leakage had occurred during external evaluation [21]. Similarly, the reported bootstrap validation of the prostate cancer nomogram did not clarify whether the full predictor-selection procedure was repeated within each resample [5]. These judgments applied to specific models and evaluations rather than uniformly to every analysis within a report. The DLBCL study was classified as Contextual because a direct prognostic comparison with expert-derived tumour burden was not established, independently of its risk-of-bias assessment [21].

Across 62 QUIPS assessment units from 33 reports, 37 were judged at high risk of bias for confounding and 50 for statistical analysis and reporting. Unadjusted HRs and outcome-driven cutoff selection were recurring limitations, reducing confidence in the independence and transportability of the reported prognostic associations. These counts refer to assessment units rather than independent studies. Across the six Form S assessment scopes, reporting was insufficient to resolve concerns about failures or missing data and selective reporting (Supplementary Appraisal). The finalized inventory includes two outcome-specific scopes for Gutsche et al. and four factor/outcome scopes for Küper et al., adopted on 15 October 2026 [19,20]. Two additional Jemaa scopes cover the full 1268-patient PFS/OS comparisons separately from its training 846 analyses [9].

## 4. Discussion

### 4.1. Principal Interpretation

This review found that AI-derived MTV and TMTV retained prognostic information in several cancer settings, with comparative evidence concentrated in lymphoma and head and neck cancer [1,3,10,27]. Selected studies reported associations between higher automated volumes and poorer outcomes, and several studies reported prognostic estimates similar to those obtained using expert-derived measurements [1,3,10,32,33]. However, these findings did not establish equivalence between methods, interchangeability of absolute volumes, or transportability of risk thresholds across populations [1,10,27]. TLG also showed prognostic associations in several studies, but its incorporation of uptake intensity requires separate interpretation from MTV and TMTV [2,4,32]. Incomplete search coverage, the absence of fully independent duplicate extraction and appraisal, and the lack of formal certainty-of-evidence grading also limit this interpretation. The findings support further evaluation of automated volumetric biomarkers for risk stratification, while their ability to improve treatment decisions and patient outcomes remains unestablished.

The distinction between prognostic association and measurement agreement is central to interpreting these findings. Automated TMTV may remain associated with outcomes even when its absolute values differ systematically from expert-derived measurements, as observed in the REMARC analysis [1]. Similar HRs obtained using method-specific cutoffs therefore do not demonstrate that the same volume threshold can be applied to both methods. Applying a common threshold, as in the PETAL analysis, provides more direct evidence about agreement in risk classification, although a threshold selected using outcomes in the evaluated cohort still requires independent validation [10]. Clinical use of automated MTV or TMTV consequently requires evidence that the measurement procedure and its associated risk threshold perform reliably in the intended population. Confidence also varied by disease and outcome. In lymphoma and head and neck cancer, threshold selection, shared-source patients and incomplete paired uncertainty limited the comparative conclusions despite some external outcome evaluations [1,3,4,10,23]. In melanoma, outcome-optimized thresholds in a small development cohort and model-specific analysis concerns limited interpretation [37]. In thymic tumours, recurrence associations were more clearly supported than OS associations, for which few deaths and intervals including the null limited inference [32]. The diagnostically mixed lung cohort and small breast cohort provided preliminary comparative evidence, with confounding and analysis limitations that precluded claims of clinical interchangeability [2,33].

### 4.2. Why Prognostic Similarity Does Not Establish Interchangeability

Systematic differences in measured MTV or TMTV may preserve the ordering of patients by tumour burden while changing their classification at a fixed volume threshold. The lower automated TMTV values observed in REMARC, despite retained prognostic associations, illustrate why these properties require separate assessment [1]. Measures of discrimination describe how well a biomarker distinguishes patients with different outcomes, but do not establish agreement in absolute volume or accuracy in estimating individual risk. Validation should therefore match the intended clinical use. Risk ranking requires assessment of discrimination, prediction of absolute risk additionally requires calibration, and threshold-based classification requires evaluation of how often patients change categories and whether those changes affect clinical decisions.

The clinical consequences of measurement error depend on how MTV or TMTV is used. A small volume difference may change risk classification when a patient’s measurement lies close to the selected threshold, whereas a larger difference may leave classification unchanged when the measurement is far from it. The PETAL analysis illustrates the value of reporting classification disagreement at a common TMTV threshold alongside prognostic associations [10]. Evaluation of automated volumetric biomarkers should therefore include absolute and relative volume differences and their effects on risk classification. For response criteria that also incorporate new-lesion status, as in PSMA PET assessment, lesion detection should be evaluated separately because total volume alone does not capture all components of the response category [39].

Comparisons with expert-derived MTV or TMTV also require a clear definition of the tissue included in each measurement. Expert contours may incorporate uptake thresholds, anatomical findings and clinical interpretation, whereas automated contours may be restricted to PET-avid disease. In the head and neck cancer non-inferiority study, the manual reference comprised clinical gross tumour volumes that included non-[^18^F]FDG-avid components, while the automated measurement represented PET-avid tumour volume [27]. The prognostic comparison therefore involved different definitions of tumour extent. Its reported shorter-term results, further qualified by the supplementary-code check, did not establish that the measurements were interchangeable or that the automated contours were suitable for radiotherapy planning. For MTV and TMTV assessment, validation should establish whether both methods quantify the same disease extent and whether any differences affect the intended clinical use.

### 4.3. Clinical Meaning Varies by Cancer and Treatment Setting

In lymphoma, whole-body TMTV quantifies the extent of metabolically active disease across multiple sites. Its prognostic relevance was observed in patients receiving first-line treatment and in those undergoing CAR-T therapy, although these populations differed substantially in treatment history and baseline risk [9,10,22]. Evidence from one setting therefore does not establish how TMTV should inform decisions in another. The GOYA analysis suggested that automated imaging measurements could add prognostic information to clinical variables, but the contribution of TMTV alone could not be isolated from the combined model [9]. Clinical integration requires evidence that automated TMTV adds useful information beyond established disease-specific risk factors in the intended treatment setting.

Head and neck cancer studies highlight the need to interpret automated MTV in relation to a specific outcome and prediction horizon. Automated tumour volume was associated with OS, while combined primary-tumour and nodal TLG provided related but distinct evidence for distant metastasis risk [3,4]. In the recurrence study, the authors reported non-inferiority at one and three years, but not at five years [27]. Same-cohort model refitting and uncertainty about the paired-difference calculation limit this inference. Failure to establish non-inferiority at five years does not itself demonstrate inferior performance. Clinical evaluation should specify the outcome and validate the prognostic procedure over the intended time horizon.

During systemic treatment, baseline TMTV, residual TMTV and change in TMTV address different prognostic questions. In advanced melanoma, baseline automated TMTV was associated with OS in patients receiving pembrolizumab, whereas a separate immune checkpoint inhibitor study found that on-treatment TMTV, but not baseline TMTV, was associated with OS [35,37]. These findings suggest that the timing of measurement may influence its prognostic relevance, although comparisons across different cohorts cannot establish the superiority of one time point. Residual volume and volume change should also be distinguished, because a low on-treatment TMTV does not necessarily indicate a large reduction from baseline. Longitudinal evaluation requires a defined assessment time, consistent imaging intervals and explicit handling of patients who progress or die before follow-up imaging. Associations observed only among patients with repeat scans may not apply to the full population starting treatment.

Evidence from prostate cancer and myeloma further shows that the interpretation of automated volume depends on the tracer and anatomical compartment being measured. PSMA-positive whole-body tumour volume and choline-defined intraprostatic volume showed prognostic associations in prostate cancer, but represented different disease extents and should not be treated as equivalent to [^18^F]FDG-derived TMTV [5,6,13,39]. In myeloma, the CASSIOPEIA analysis found prognostic associations for medullary TMTV but not focal-lesion TMTV, indicating that the scope of measurement can influence its clinical relevance [41]. Exploratory methionine and CXCR4 studies provided additional evidence for tracer-specific measures, without establishing their interchangeability with [^18^F]FDG-derived volumes [7,8]. These findings broaden the context for automated volumetric assessment while supporting separate interpretation by tracer, anatomical scope and treatment setting. Spatial PET/CT features may provide complementary prognostic information. In locally advanced NSCLC, Ji et al. reported associations of metabolic habitat and hotspot-location measures with progression, supporting evaluation of spatial heterogeneity alongside global tumour burden [43].

### 4.4. External Validation Must Include the Full Prognostic Procedure

External validation of automated MTV or TMTV requires separate consideration of the segmentation model and the prognostic rule applied to its measurements. A pretrained model may generate volumes in an independent imaging cohort, but selecting a cutoff using outcomes from that cohort makes the prognostic analysis part of model development. In the DLBCL study with a 117-patient external cohort, the reported methods did not establish whether the TMTV cutoff had been fixed before prognostic evaluation [24]. This uncertainty limits conclusions about threshold transportability without negating the observed prognostic association. Similarly, multicentre training does not by itself establish external validation when the test cohort comes from an institution that contributed training data, as in the lung cancer study [2]. Validation claims should therefore specify which components were fixed in advance and which were developed or modified using the evaluation cohort.

A clinically informative external evaluation should apply a prespecified measurement procedure and prognostic rule to an independent population representative of the intended clinical setting. Performance should be assessed without outcome-driven changes to the model or cutoff, with uncertainty reported for discrimination, calibration where absolute risk is predicted, and classification at clinically relevant thresholds. The lung cancer feasibility study illustrates why the composition of the evaluation cohort matters. Discrimination was limited in the complete validation cohort, and higher AUCs in exploratory stage-balanced subsamples did not establish better performance in the full population [31]. Such subgroup analyses may help explain variation in performance, but cannot replace evaluation in the population in which automated MTV is intended to support clinical decisions.

Manual review and correction form part of the measurement procedure and should be included in its validation. When automated contours are edited before MTV or TMTV calculation, the resulting prognostic performance describes the combined contribution of the algorithm and human intervention. Studies should therefore specify whether measurements were used directly, reviewed without modification or corrected, and report the frequency and extent of correction together with the total review time. Excluding scans for which automated measurement fails may overestimate performance in routine practice. Evaluation should account for all attempted scans, explain how failures were handled and assess whether cases requiring correction differ systematically from those processed successfully.

### 4.5. Statistical Requirements for Comparative Claims

Comparative claims require direct estimation of the difference between automated and expert-derived prognostic results. Similar HRs or overlapping confidence intervals do not establish equivalent performance. When both measurements are evaluated in the same patients, their estimates are statistically dependent because they share outcomes and correlated predictors. Comparisons should account for this dependence, for example by resampling patients and refitting both models within each bootstrap sample to estimate uncertainty in the paired difference. Correlation between measured volumes alone cannot provide the covariance between the fitted prognostic coefficients. If equivalence or non-inferiority is the intended claim, the analysis should also specify a clinically justified margin in advance, and estimate uncertainty in the paired difference. The head and neck study specified margins, but its supplied calculation did not explicitly demonstrate this paired uncertainty [27].

The magnitude of an HR also depends on how MTV or TMTV is expressed in the analysis. An HR per unit increase in volume, an HR for log-transformed volume and an HR comparing high- and low-volume groups describe different contrasts and cannot be compared directly as measures of biomarker performance. Selecting separate outcome-based cutoffs for automated and expert measurements further changes the groups being compared, as in the REMARC analysis [1]. Evaluation of clinical substitution therefore requires a common decision rule or a prespecified approach to recalibrating the threshold, followed by independent validation. In the projection-based lymphoma study, uncertainty about the predictor coding or transformation underlying the reported HRs prevented their inclusion in quantitative synthesis [23]. Clear reporting of units, transformations, cutoff selection and covariate adjustment is essential for interpretable comparisons.

A prognostic association does not establish that automated MTV or TMTV improves prediction beyond information already available to clinicians. When a model combines imaging measurements with clinical or molecular variables, its overall performance cannot be attributed to the volumetric biomarker alone, as illustrated by the combined models evaluated in DLBCL [9,21]. The additional contribution of MTV or TMTV should be assessed by comparing otherwise comparable models with and without the volume measure in independent data. Even an improvement in prediction does not demonstrate that treatment decisions guided by the biomarker improve patient outcomes. That claim requires prospective evaluation of a defined decision strategy. The evidence synthesized here therefore supports further assessment of automated MTV and TMTV for risk stratification, but does not justify treatment changes solely on the basis of their association with survival. Habitat and metabolic-hotspot analyses may address complementary spatial information, but manual tumour delineation followed by Otsu thresholding is distinct from AI-derived PET tumour-burden measurement [43]. Response-model comparisons and progression associations in such a report do not alone establish incremental prognostic value over MTV.

### 4.6. Strengths and Limitations of the Review

A strength of this review is its distinction between direct prognostic comparisons with expert-derived measurements and contextual evidence for AI-derived PET tumour-burden biomarkers. Tracking shared patient cohorts across reports helped avoid interpreting repeated analyses of the same population as independent validation. Recording analysis-specific sample sizes, predictor transformations and threshold definitions also clarified which comparisons addressed similar clinical questions and which required separate interpretation. The synthesis maintained MTV and TMTV as its central focus while distinguishing TLG and volumes defined by other tracers or anatomical compartments. This approach allowed the evidence to be considered across cancer settings without assuming that all AI-derived PET measurements represented the same biomarker.

The conclusions are limited by heterogeneity in patient populations, treatment settings, measurement definitions and prognostic analyses. Incomplete reporting of uncertainty in paired comparisons, outcome-driven cutoff selection and limited external prognostic validation prevented a pooled estimate of the difference between automated and expert-derived measurements. Repeated use of public datasets and trial cohorts also meant that the number of reports exceeded the number of independent patient populations. Missing event counts, follow-up details and confidence intervals in some reports restricted assessment of precision and generalizability. One proceedings report was available for assessment only at abstract level, limiting evaluation of its methods and findings [25]. These limitations support cautious interpretation of the observed prognostic associations and preclude a general claim of equivalence between automated and expert-derived MTV or TMTV.

The search strategy also has limitations. Searches of PubMed and Embase were supplemented by Google Scholar screening and backward and forward citation searching, but eligible studies may still have been missed. Restriction to English-language reports and the conditional exclusion of conference records in Embase may have introduced language and publication-type bias. One proceedings report identified through supplementary searching was retained as Contextual evidence at the abstract level, creating a difference in the availability of evidence across search routes [25]. Reproducibility of the Google Scholar search was further limited because the retained search record did not contain the exact executed query or personalization settings. These limitations should be considered when interpreting the completeness of the evidence identified. Web of Science, Scopus, and IEEE Xplore were not searched because institutional subscription access was unavailable when the review was initiated. This restriction may have reduced coverage of technical and multidisciplinary literature, particularly conference proceedings. The PubMed and Embase searches and supplementary searching do not fully resolve this coverage limitation. The revision audit included conference records but retained publication-type exclusions; it did not provide a sensitivity synthesis including conference abstracts. Revision follow-up also retained the original temporal eligibility boundary, so publications appearing after 9 September 2026 may have been missed.

The appraisal process also limits the review. Initial assessments used a review-specific framework that differed from the planned tool-specific approach. The original extraction and appraisals underwent second-researcher verification and final adjudication, but these procedures did not constitute independent duplicate extraction and assessment. Structural correspondence with the complete official PROBAST + AI form was checked during revision, but source-level validity of every response remains unverified, and the initial appraisal grid was retained separately rather than treated as a validated-tool assessment. The revision-stage applicability addendum clarifies the intended populations, predictors and outcomes, but does not resolve incomplete source-level verification. Following AI-assisted review, Sang Jun Byun independently reviewed the additional and changed revision materials, and Byungwook Choi made the final decisions. These procedures leave scope for reviewer judgment and assessment error. Narrative synthesis also involves judgment in selecting and interpreting representative findings. To make that interpretation transparent, we reported the comparator, analysis population and relevant limitations alongside the prognostic results. The disease- and outcome-specific confidence statements were qualitative interpretations of the existing extraction and appraisal, not formal certainty grades or independent duplicate appraisals from scratch. They should therefore not be interpreted as establishing a formally graded level of certainty for any clinical application. Core membership was not treated as a low-risk-of-bias or high-certainty designation.

### 4.7. Priorities for Clinical Evaluation

Future studies should define the intended clinical use of automated MTV or TMTV before selecting the validation design. Replacement of expert quantification requires comparisons of measurements and prognostic performance in the same patients, including scans requiring correction or yielding no usable automated result. For risk prediction, a fixed measurement procedure and prognostic model should undergo independent testing against an appropriate clinical reference model. Treatment adaptation poses a further question of whether using MTV or TMTV to guide a defined treatment decision improves patient outcomes or reduces treatment burden without compromising disease control. This question requires prospective evaluation of a prespecified decision strategy. Aligning each study with a specific use would make its findings more directly applicable to clinical practice.

Clear reporting is essential for interpreting and reproducing these evaluations. The measurement description should identify the tracer, anatomical coverage, segmentation method, manual correction rules and model version, together with the separation between development and evaluation datasets. Prognostic analyses should specify the predictor units and transformations, threshold derivation, outcome definition and the time point from which follow-up begins. Sample sizes, event counts, follow-up duration and covariate adjustment should accompany the estimates, with uncertainty in paired differences reported for direct comparisons. Naming shared datasets and identifying overlapping cohorts would also help distinguish independent validation from repeated analysis of the same patients. These reporting priorities address the main barriers encountered in this review and align with guidance for imaging AI and clinical prediction models [16,17].

## 5. Conclusions

AI-derived MTV and TMTV showed prognostic associations in several cancers, supporting further investigation of automated PET tumour-burden measurement for risk stratification. Although selected studies reported results similar to expert measurements, interchangeability and benefits for treatment decisions remain unproven. Findings for TLG and other tracer-defined volumes require separate interpretation. Direct comparisons in the same patients and independent validation of prespecified thresholds are needed to establish clinical applicability. Given limitations in search coverage, review procedures and certainty assessment, further prospective evaluation is needed to determine whether these biomarkers improve clinical decisions and patient outcomes.

## Figures and Tables

**Table 1:** Characteristics of the 35 included reports.

Report/Tier	Design and Clinical Setting	Prognostic Sample, *n*	Tracer and Burden Measure	AI Method and Comparator	Outcomes and Validation
**Lymphoma**					
Nikulin et al., 2026 [10] Core	Trial secondary analysis; aggressive non-Hodgkin lymphoma, PETAL	534	[^18^F]FDG; baseline TMTV	nnU-Net ensemble; manual TMTV	PFS; internal outcome analysis; external segmentation test only
Wang et al., 2025 [21] Contextual	Retrospective multicentre; DLBCL, R-CHOP-based treatment	580 internal; 194 external; 90 R-CHOP-X	[^18^F]FDG; baseline TMTV, TLG	Pretrained nnU-Net; clinical/molecular models; no expert-burden prognostic comparator	PFS, OS; external prognostic evaluation with fixed cutoffs
Galli et al., 2025 [22] Contextual	Retrospective single-centre; aggressive B-cell lymphoma, CAR-T therapy	37	[^18^F]FDG; baseline TMTV, TLG	Lesion Scout/Auto ID, physician review; no expert-burden prognostic comparator	PFS; same-cohort cutoff selection and modelling
Jemaa et al., 2022 [9] Core	Retrospective trial imaging analysis; DLBCL, GOYA	1268: 846 dev.; 422 holdout	[^18^F]FDG; baseline TMTV; combined score	Automated TMTV; same-patient manual TMTV comparison	PFS, OS; full-cohort comparison; internal holdout for risk model
Girum et al., 2022 [23] Core	Retrospective two-cohort analysis; DLBCL, REMARC and LNH073B	287 REMARC; 95 LNH073B	[^18^F]FDG; 2D tumour-area and dissemination surrogates	PET maximum-intensity-projection CNN; expert 3D TMTV/dissemination	PFS, OS; independent external cohort
Jiang et al., 2022 [24] Contextual	Retrospective dual-centre; DLBCL	117 external outcome cohort	[^18^F]FDG; baseline TMTV	3D nnU-Net; manual measurement agreement only	PFS, OS; external imaging cohort, but cutoff selected in that cohort
Capobianco et al., 2021 [1] Core	Retrospective trial ancillary analysis; DLBCL, REMARC	280	[^18^F]FDG; baseline TMTV	PARS; expert TMTV	4-year PFS, OS; pretrained model application; cutoffs selected in outcome cohort
Luo et al., 2026 [25] Contextual	Retrospective single-cohort study; limited-stage follicular lymphoma; proceedings report	82	[^18^F]FDG; PET burden features	Threshold/AI consensus and union masks; no expert-burden prognostic comparator	Progression; internal cross-validation; abstract-level assessment
Le Goff et al., 2023 [26] Contextual	Single-centre observational; untreated LBCL, Créteil	102 PET/112 enrolled	[^18^F]FDG; baseline TMTV	PAIRE v1.0.0; visual/CT checks; no expert prognostic comparator	PFS; outcome-optimized 200 mL cutoff; two QUIPS scopes finalized
**Head and neck cancer**					
Kovacs et al., 2026 [27] Core	Retrospective temporal evaluation; head and neck cancer	540	[^18^F]FDG; PET-avid tumour volume	nnU-Net; manual clinical gross tumour volume	Locoregional failure, distant metastasis; separately refitted models evaluated in the same cohort
Kovacs et al., 2025 [28] Contextual	Retrospective external cohort; head and neck cancer after radiotherapy or chemoradiotherapy	50	[^18^F]FDG; change in PET-avid tumour volume	Fixed nnU-Net applied without retraining; no expert-burden prognostic comparator	Locoregional control; external cohort; cohort-specific cutoff
Lv et al., 2025 [29] Contextual	Retrospective single-centre; non-metastatic nasopharyngeal carcinoma	83	[^18^F]FDG; primary-tumour MTV, TLG	PARS with review/correction; manual agreement assessment only	PFS, OS; same-cohort prognostic modelling
Zschaeck et al., 2025 [4] Core	Retrospective multicentre; oropharyngeal carcinoma, curative treatment	366 exploratory; 65 validation	[^18^F]FDG; primary-plus-nodal TLG	CNN ensemble; manual TLG in validation patients	Distant metastasis; fixed cutoff tested in independent cohort
Nikulin et al., 2023 [3] Core	Retrospective multicentre; head and neck squamous cell carcinoma	585 main; 142 external	[^18^F]FDG; primary-plus-nodal tumour burden	Residual 3D U-Net with attention; manual tumour burden	OS; external cohort with cutoff fixed from main cohort
Leung et al., 2024 [30] Contextual	Retrospective; head and neck cancer outcome subset of multi-cancer study	74	[^18^F]FDG for outcome analysis; MTV	DeepSSTL with nnU-Net; no expert-burden prognostic comparator	OS; development/evaluation in same cohort
**Lung cancer**					
Borrelli et al., 2022 [2] Core	Retrospective two-centre; suspected lung cancer, mixed final diagnoses	106 held-out patients	[^18^F]FDG; TLG	RECOMIA 3D U-Net; manual TLG	OS; patient-level internal holdout
Nafeli et al., 2026 [31] Contextual	Retrospective two-cohort feasibility study; non-small cell lung cancer	150 dev.; 89 test	[^18^F]FDG; MTV, TLG and other PET features	LIONZ segmentation; PET prognostic models; no expert-burden prognostic comparator	1-year mortality; separate study cohort at same imaging centre
**Thymic epithelial tumours**					
Han et al., 2022 [32] Core	Retrospective single-centre; surgically resected thymic epithelial tumours	114	[^18^F]FDG; MTV, TLG	Quasi-3D residual U-Net; manual MTV/TLG	Freedom from recurrence, OS; internal test folds; no independent outcome cohort
**Breast cancer**					
Weber et al., 2021 [33] Core	Retrospective single-centre; predominantly advanced breast cancer	50	[^18^F]FDG; whole-body and regional MTV	PARS trained on lymphoma/lung cancer; manual MTV	OS; external cancer-type application; outcome analysis in same cohort
Liang et al., 2026 [34] Contextual	Retrospective single-centre; non-metastatic breast cancer	1210 overall; OS test 610; DFS test 570	[^18^F]FDG; pretreatment primary-plus-nodal MTV, TLG	STU-Net; no expert-burden prognostic comparator	OS, DFS; internal test; validation-set median thresholds
**Melanoma**					
Sachpekidis et al., 2026 [35] Contextual	Retrospective analysis of prospective cohort; metastatic melanoma, immunotherapy	43	[^18^F]FDG; serial TMTV, TLG	Ensemble segmentation and classification with expert correction; no expert-burden prognostic comparator	OS; same-cohort longitudinal analysis
Sachpekidis et al., 2026 [36] Contextual	Prospective single-centre exploratory cohort; uveal melanoma, tebentafusp	15	[^18^F]FDG; baseline and 3-month TMTV, TLG	RECOMIA; no expert-burden prognostic comparator	OS; same-cohort median-split analysis
Dirks et al., 2023 [37] Core	Retrospective single-centre; advanced melanoma, pembrolizumab	69 dev.; 31 holdout	[^18^F]FDG; baseline TMTV, TLG	nnU-Net; manual burden in development cohort	OS, PFS; internal holdout for combined OS model
**Prostate cancer**					
Zang et al., 2026 [5] Contextual	Retrospective single-centre; metastatic castration-resistant prostate cancer, lutetium-177 PSMA therapy	107	[^68^Ga]Ga-PSMA-11; baseline TTV	Multi-task nnU-Net; no expert-burden prognostic comparator	OS; internal bootstrap validation
Beintner-Skawran et al., 2025 [38] Contextual	Retrospective multicentre; metastatic prostate cancer, taxane chemotherapy	128	[^68^Ga]Ga-PSMA-11; baseline and change in TTV	DeepPSMA with limited manual adjustment; no expert-burden prognostic comparator	OS; same-cohort cutoff selection and modelling
Kendrick et al., 2023 [39] Core	Trial secondary analysis; biochemically recurrent prostate cancer	199 manual; nested AI subset 74	[^68^Ga]Ga-PSMA-11; TTV change and RECIP	3D U-Net cascade/nnU-Net; manual TTV/RECIP	OS; nested internal test subset; unequal comparison samples
Kendrick et al., 2022 [40] Contextual	Prospective registered cohort; retrospective AI/prognostic analysis; recurrent prostate cancer	72	[^68^Ga]Ga-PSMA-11; baseline tumour volume and uptake	Cascaded 3D nnU-Net; no expert-burden prognostic comparator	OS; held-out patients from same registered cohort
Polymeri et al., 2021 [6] Contextual	Retrospective independent-institution cohort; high-risk prostate cancer	285	[^18^F]choline; intraprostatic uptake-defined volume	AI prostate segmentation plus uptake threshold; no expert-burden prognostic comparator	Prostate cancer-specific survival; independent-institution application
Polymeri et al., 2020 [13] Contextual	Retrospective model development and external assessment; hormone-naive prostate cancer with bone metastases	43	[^18^F]choline; intraprostatic uptake-defined volume	Prostate CNN plus uptake threshold; two expert readers	OS; independent-institution application
Küper et al., 2024 [20] Contextual	Retrospective paired PET; non-LuPSMA prostate cancer	73; 26 deaths	[68Ga]Ga-PSMA-11; body-weight-adjusted total/regional volume and change	MICIIS; neural-network masks inferred from cited method; manual additions	OS associations; no paired expert prognostic comparison
**Multiple myeloma**					
Jamet et al., 2026 [41] Contextual	Prospective ancillary study/secondary analysis; newly diagnosed myeloma, CASSIOPEIA	195; survival-ML complete cases 187	[^18^F]FDG; medullary and focal-lesion TMTV	CT bone/liver segmentation plus PET quantification; no expert-burden prognostic comparator	PFS, OS; internal nested cross-validation of prognostic ensemble
Xue et al., 2026 [8] Contextual	Retrospective single-centre; relapsed/refractory myeloma, CXCR4-directed therapy	22 patients; 24 treatment cycles	[^18^F]FDG and [^68^Ga]Ga-pentixafor; lesion volume, TLG	nnU-Net with registration and anatomical masks; no expert-burden prognostic comparator	OS; exploratory same-cohort analysis; patient/cycle ambiguity
Ye et al., 2026 [7] Contextual	Prospective single-centre; newly diagnosed myeloma	37 PFS-evaluable patients	[^11^C]methionine and [^18^F]FDG; bone marrow MTV, TLG	AI skeleton segmentation plus PET quantification; comparison across tracers	PFS; same-cohort threshold selection; no external validation
Sachpekidis et al., 2024 [42] Contextual	Prospective single-centre observational cohort; newly diagnosed myeloma	44	[^18^F]FDG; bone marrow MTV, TLG	CT-organ CNN plus PET thresholds; no expert-burden prognostic comparator	PFS, OS; same-cohort exploratory analyses
**Glioma**					
Gutsche et al., 2023 [19] Core	Retrospective glioma response cohort; adjuvant temozolomide	33 reported; risk sets in the source report total 32	[18F]FET; MTV percentage change	nnU-Net; physician MTV in the same patients	OS and PFS; unadjusted; response-cohort independence unverified

Reports are grouped by clinical population and comprise 13 Core and 22 Contextual reports from 31 catalogue families. Sample sizes refer to prognostic analyses, not segmentation training datasets; multiple samples within or across reports must not be summed as independent patients. Shared cohort families include REMARC (Capobianco [1] and Girum [23]), head-and-neck source datasets (Nikulin [3], Zschaeck [4] and Leung [30]), and the registered prostate cohort (Kendrick [39,40]); overlap is not necessarily complete. Borrelli [2] includes mixed diagnoses among patients investigated for suspected lung cancer. Leung [30] is classified by its head-and-neck outcome subset. Core and Contextual indicate roles in the synthesis, not risk of bias. Detailed extraction and source locations are provided in Supplementary Data. AI, artificial intelligence; CAR-T, chimeric antigen receptor T-cell; CNN, convolutional neural network; CXCR4, C-X-C chemokine receptor 4; dev., development; DFS, disease-free survival; DLBCL, diffuse large B-cell lymphoma; FDG, fluorodeoxyglucose; ML, machine learning; MTV, metabolic tumour volume; OS, overall survival; PARS, PET-Assisted Reporting System; PET, positron emission tomography; PFS, progression-free survival; PSMA, prostate-specific membrane antigen; RECIP, Response Evaluation Criteria in PSMA PET/CT; R-CHOP, rituximab, cyclophosphamide, doxorubicin, vincristine and prednisone; TLG, total lesion glycolysis; TMTV, total metabolic tumour volume; TTV, total tumour volume.

**Table 2:** Representative prognostic comparisons from the 13 Core reports.

Report and Outcome Sample	Outcome and Predictor	AI Versus Expert Result	Interpretation
Nikulin et al., 2026 [10]; PETAL aggressive non-Hodgkin lymphoma; *n* = 534	PFS; TMTV > 210.2 versus ≤210.2 mL	HR 3.7 versus 3.5; 95% CIs not reported	The cutoff was optimized using manual TMTV and applied to both methods. Uncertainty in the paired HR difference was not reported.
Kovacs et al., 2026 [27]; head and neck cancer; *n* = 540	1-year locoregional failure; models incorporating AI-derived PET-avid volume or manual clinical GTV	AUC 77.3% (72.2–82.4%) versus 77.0% (71.9–82.0%)	Authors reported non-inferiority at 1 and 3 years, not at 5 years. Models were refitted and scored in the same cohort; paired-difference uncertainty was not explicitly demonstrated by the supplied calculation. Manual and AI measurements represented different tumour extents.
Zschaeck et al., 2025 [4]; oropharyngeal carcinoma; external validation *n* = 65	Distant metastasis; combined primary-tumour and nodal TLG > 290 versus ≤290	HR 3.1 versus 3.47; 95% CIs not reported	The cutoff was derived in the exploratory cohort and applied unchanged in validation. Analyses were univariable, and TLG units were inconsistently reported. Cohorts overlapped with those of Nikulin et al. [3].
Dirks et al., 2023 [37]; advanced melanoma treated with pembrolizumab; development *n* = 69	OS; TMTV > 90 versus ≤90 mL	HR 14.3 (5.72–35.8) versus 12.2 (4.95–29.8)	These univariable HRs were estimated in the development cohort using a cutoff optimized on manual TMTV. Validation in 31 separate patients assessed a combined prognostic model, not these TMTV-specific HRs.
Kendrick et al., 2023 [39]; biochemically recurrent prostate cancer; AI *n* = 74/manual *n* = 199	OS; RECIP 1.0 progression versus non-progression on PSMA PET/CT	HR 3.75 (1.23–11.47) versus 3.78 (1.96–7.28)	The AI subset was nested within the larger manual cohort, so these HRs do not provide a same-patient comparison. RECIP combines changes in PSMA-positive tumour volume with new-lesion status.
Nikulin et al., 2023 [3]; head and neck squamous cell carcinoma; external OS analysis *n* = 142	OS; primary-tumour and nodal TTB above versus at or below the fixed manual-derived cutoff	HR 1.9 versus 1.8; 95% CIs not reported	The median manual TTB in the main cohort was applied as a common cutoff in the external cohort. Uncertainty in the paired HR difference was not assessed. Cohorts overlapped with those of Zschaeck et al. [4].
Girum et al., 2022 [23]; DLBCL; REMARC *n* = 287/LNH073B *n* = 95	PFS; AI-derived two-dimensional tumour area versus expert-derived three-dimensional TMTV	REMARC AUC 0.65 versus 0.67; LNH073B AUC 0.66 versus 0.62	The AI measure was an area-based surrogate for TMTV. AUCs are presented descriptively; the predictor scale underlying the HRs could not be verified. The REMARC cohort overlapped with that of Capobianco et al. [1].
Han et al., 2022 [32]; surgically resected thymic epithelial tumours; *n* = 114	Freedom from recurrence; per unit increase in log_10_-transformed MTV	HR 2.11 (1.09–4.06) versus 2.43 (1.33–4.45)	Both measurements were associated with recurrence in univariable analyses of the same patients. No paired test of the HR difference or formal equivalence analysis was reported.
Borrelli et al., 2022 [2]; suspected lung cancer with mixed final diagnoses; test cohort *n* = 106	OS; per unit increase in log_10_(TLG + 1)	HR 1.64 (1.21–2.21) versus 1.54 (1.14–2.07)	These univariable associations were evaluated in the same held-out patients. The test institution also contributed training data. Uncertainty in the paired HR difference was not reported.
Weber et al., 2021 [33]; predominantly advanced breast cancer; *n* = 50	OS; per unit increase in log_2_(whole-body MTV + 1)	HR 1.275 (1.122–1.448) versus 1.438 (1.208–1.713)	The model was trained on lymphoma and lung cancer and applied without breast-cancer-specific training. Both measurements were associated with OS in univariable analyses, but the small cohort limited generalizability.
Capobianco et al., 2021 [1]; DLBCL; REMARC *n* = 280	4-year PFS; TMTV, with HR cutoffs of 171 cm^3^ for AI and 242 cm^3^ for expert measurements	AUC 0.63 versus 0.69; HR 2.3 (1.5–3.6) versus 2.6 (1.6–4.1)	The HRs used different cutoffs optimized within the same cohort and therefore do not represent a comparison at a common threshold. The REMARC cohort overlapped with that of Girum et al. [23].
Gutsche et al., 2023 [19]; glioma response cohort; 33 reported/32 at risk	OS and PFS; MTV change ≤ 0% versus >0%	OS: both *p* < 0.05; PFS: AI *p* = 0.39, physician *p* = 0.12	Unadjusted log-rank analyses; no paired difference test or HR/CI; not equivalence evidence.
Jemaa et al., 2022 [9]; GOYA full evaluable cohort; *n* = 1268	PFS/OS; median and Q3 TMTV splits	PFS AI/manual HR: 1.70/1.76 (median), 1.94/1.96 (Q3); OS: 1.72/1.83, 2.03/2.11	Same patients; actual cutoff alignment and adjustment unspecified. Outside aligned set; no equivalence inference.

Results are presented as AI-derived versus expert-derived measurements; parentheses indicate source-reported 95% confidence intervals. Each row summarises selected findings from one report, and patient cohorts may overlap across reports. Similar estimates do not establish equivalence or interchangeability between measurement methods. In Nikulin et al. [3], TTB denotes the sum of primary-tumour and nodal MTV. Source locations and further extraction details are provided in Supplementary Data. AI, artificial intelligence; AUC, area under the receiver operating characteristic curve; CI, confidence interval; DLBCL, diffuse large B-cell lymphoma; GTV, gross tumour volume; HR, hazard ratio; MTV, metabolic tumour volume; OS, overall survival; PET/CT, positron emission tomography/computed tomography; PFS, progression-free survival; PSMA, prostate-specific membrane antigen; RECIP, Response Evaluation Criteria in PSMA PET/CT; TLG, total lesion glycolysis; TMTV, total metabolic tumour volume; TTB, total tumour burden.

**Table 3:** Classification of 69 previously documented comparisons.

Comparison Category	Comparisons	Interpretation
Aligned burden comparison	23	Tumour burden was compared in the same patients using aligned predictor contrasts or discrimination metrics. These comparisons do not establish equivalence or automatically qualify for pooling.
Nested unequal samples	2	Automated measurements were evaluated in a subset of 74 patients, whereas manual measurements were evaluated in the parent cohort of 199 patients. The reported estimates therefore do not provide a paired comparison in the same patients.
Different segmentation targets	14	Automated PET-avid gross tumour volume was compared with manually delineated clinical gross tumour volume. Similar prognostic performance does not establish agreement between the tumour contours.
Cross-tracer comparison	2	Methionine and [^18^F]FDG measurements were compared using automated segmentation. These comparisons assess differences between tracers rather than between automated and expert measurements.
Area versus volume surrogate	8	Two-dimensional tumour area was compared with three-dimensional tumour volume. The predictor scales underlying the HRs could not be verified, precluding their use in quantitative synthesis. AUCs were retained for descriptive comparison.
Dissemination surrogate	8	A two-dimensional dissemination surrogate was compared with three-dimensional Dmax. Both describe disease dissemination rather than tumour volume. The predictor scales underlying the HRs could not be verified, precluding their use in quantitative synthesis.
Method-specific thresholds	2	Automated and expert measurements were evaluated in the same patients using separately optimized, outcome-based cutoffs. The resulting HRs compare different risk groups and do not assess performance at a common threshold.
Uptake-only comparison	4	SUVmax or SUVmean describes tracer uptake intensity rather than tumour volume.
Anatomical gland volume	2	Anatomical prostate-gland volume was measured rather than lesion-defined MTV.
Gland uptake-weighted burden	2	Prostate-gland uptake-weighted burden was evaluated as contextual evidence. This measure differs from whole-body lesion TMTV.
Uptake fraction	1	Relative intraprostatic uptake fraction was evaluated as contextual evidence and does not measure tumour volume.
Different AI biomarkers	1	AI-derived TMTV was compared with AI-derived TLG in Wang et al. [21]. This comparison was excluded from the AI-versus-expert synthesis.

Counts refer to individual comparisons, not independent reports or cohorts. The categories distinguish comparisons of tumour burden from those involving different segmentation targets, tracers or biomarkers. The 23 aligned burden comparisons do not establish equivalence between methods and are not automatically eligible for quantitative pooling. One comparison in Wang et al. [21] involved AI-derived total metabolic tumour volume and total lesion glycolysis, rather than AI-derived and expert-derived measurements, and was excluded from the AI-versus-expert synthesis. Details of all comparisons are provided in Supplementary Data. AI, artificial intelligence; AUC, area under the receiver operating characteristic curve; Dmax, maximum distance between lesion centroids; FDG, fluorodeoxyglucose; HR, hazard ratio; MTV, metabolic tumour volume; PET, positron emission tomography; SUVmax, maximum standardized uptake value; SUVmean, mean standardized uptake value; TLG, total lesion glycolysis; TMTV, total metabolic tumour volume.

## Data Availability

The extracted study-level data and comparative analyses are provided in Supplementary Data, with source locations and DOI identifiers for verification. Risk-of-bias judgments and restrictions on quantitative synthesis are summarized in Supplementary Appraisal. Detailed judgments, supporting rationales, source locations and reviewer confirmation records are provided in the accompanying Supplementary Assessment Details workbook and JSON file. No individual patient data were obtained.

## Supplementary Materials

The following supporting information can be downloaded at: https://www.mdpi.com/article/10.3390/diagnostics16213564/s1, Supplementary Methods describes the database search strategies, study selection counts, comparison definitions and methodological deviations. Supplementary Data contains the catalogue of 35 reports, links between shared cohorts, extracted effect estimates and details of the comparative analyses, provided as an Excel workbook with a machine-readable JSON companion. Supplementary Appraisal presents the QUIPS and Form S assessments and restrictions on the use of individual estimates in quantitative synthesis. Current appraisal tables supersede historical entries retained for provenance. Supplementary Data contains 361 effect records, including the adopted revision additions for Liang, Gutsche, Küper and Le Goff. Supplementary Assessment Details provides the finalized QUIPS and Form S domain judgments and PROBAST + AI records, supporting rationales, source locations, reviewer confirmation records, restrictions on the use of effect estimates, and assessment history as an XLSX workbook and a JSON companion. Supplementary Excluded Reports lists the 69 unique reports excluded after full-text assessment, with titles, DOI identifiers and reasons. The PRISMA 2020 Checklist identifies reporting locations and remaining reporting limitations. Supplementary Methods S9 includes the 23 aligned comparison records, comparison conditions, cohort-dependence and validation qualifications, and the restricted pooling assessment (Tables S1, S2 and S2a). The supplementary bibliography and methodological sources are included in the reference list [1,2,3,4,5,6,7,8,9,10,11,12,13,14,15,16,17,18,19,20,21,22,23,24,25,26,27,28,29,30,31,32,33,34,35,36,37,38,39,40,41,42,43], together with additional sources cited in Supplementary Methods [44,45,46,47,48,49,50,51,52,53,54,55,56,57,58,59,60,61,62,63,64,65,66,67,68,69,70,71,72].
