# Supplementary material for: Prognostic Evidence for Artificial Intelligence-Derived PET Tumour-Burden Biomarkers in Cancer: A Systematic Review
Source: Diagnostics (Basel). 2026 Oct 29;16(21):3564. doi: 10.3390/diagnostics16213564 (PMC13762039; doi:10.3390/diagnostics16213564)
Supplement: Supplementary file 1 [file diagnostics-16-03564-s001.zip › PRISMA 2020 Checklist.pdf]

PRISMA 2020 Checklist

Locations refer to manuscript sections and named supplementary files. Partial reporting and methods not performed are stated explicitly.

Prognostic evidence for artificial intelligence-derived PET tumour-burden biomarkers in cancer: a systematic review

Section and Topic	Item #	Checklist item	Location where item is reported
**TITLE**			
Title	1	Identify the report as a systematic review.	Title.
**ABSTRACT**			
Abstract	2	See the PRISMA 2020 for Abstracts checklist.	Abstract. Reports objectives, eligibility, search dates, synthesis, included reports and cohort families, main findings, limitations, interpretation and registration. Funding is reported separately in the Funding section.
**INTRODUCTION**			
Rationale	3	Describe the rationale for the review in the context of existing knowledge.	Section 1, Introduction, paragraphs 1–6.
Objectives	4	Provide an explicit statement of the objective(s) or question(s) the review addresses.	Section 1, final paragraph.
**METHODS**			
Eligibility criteria	5	Specify the inclusion and exclusion criteria for the review and how studies were grouped for the syntheses.	Sections 2.2 and 2.6; Supplementary Methods S4–S5.
Information sources	6	Specify all databases, registers, websites, organisations, reference lists and other sources searched or consulted to identify studies. Specify the date when each source was last searched or consulted.	Section 2.3; Supplementary Methods S1–S4, S24–S25. PubMed and Embase were searched on 29 August 2026; supplementary searching closed on 9 September 2026; the revision export is dated 13 October 2026. Exact individual supplementary-search dates are not fully specified. Web of Science, Scopus and IEEE Xplore were unavailable through institutional subscription from the outset; this is a historical coverage limitation, not a pending search. The original 9 September temporal eligibility boundary was retained; the revision export was not a comprehensive search update through 15 October.
Search strategy	7	Present the full search strategies for all databases, registers and websites, including any filters and limits used.	Supplementary Methods S2–S4 and S24. Original database strategies and available citation-search procedures are supplied. Exact revision-query reproducibility is limited to archived source records. The exact Google Scholar query and account/personalization settings were not retained. These limitations are disclosed.
Selection process	8	Specify the methods used to decide whether a study met the inclusion criteria of the review, including how many reviewers screened each record and each report retrieved, whether they worked independently, and if applicable, details of automation tools used in the process.	Section 2.3; Supplementary Methods S6 and S24. Two reviewers independently screened the original 3188 titles/abstracts. Hae Won Kim verified original full-text decisions, Sang Jun Byun checked Core reports, and Byungwook Choi finalized decisions. Following AI-assisted review, Sang Jun Byun independently reviewed the additional and changed revision materials, and Byungwook Choi made the final decisions. Independent duplicate screening of the complete revision set was not performed.

PRISMA

PRISMA 2020 Checklist

Section and Topic	Item #	Checklist item	Location where item is reported
Data collection process	9	Specify the methods used to collect data from reports, including how many reviewers collected data from each report, whether they worked independently, any processes for obtaining or confirming data from study investigators, and if applicable, details of automation tools used in the process.	Section 2.4; Supplementary Methods S6 and S24–S25. Human verification of AI-assisted extraction is described. Following AI-assisted review, Sang Jun Byun independently reviewed the additional and changed revision materials, and Byungwook Choi made the final decisions. This does not establish independent duplicate extraction. No investigator contact to obtain additional data is reported.
Data items	10a	List and define all outcomes for which data were sought. Specify whether all results that were compatible with each outcome domain in each study were sought (e.g. for all measures, time points, analyses), and if not, the methods used to decide which results to collect.	Sections 2.4 and 2.6; Supplementary Methods S6; Supplementary Data, outcome and effect records. Eligible prognostic results were sought as comprehensively as possible. Outcome-specific definitions are retained; an exhaustive collection of every eligible result is not claimed, and a detailed rule for selecting among multiple results is not specified.
	10b	List and define all other variables for which data were sought (e.g. participant and intervention characteristics, funding sources). Describe any assumptions made about any missing or unclear information.	Section 2.4; Supplementary Methods S7–S8; Supplementary Data.
Study risk of bias assessment	11	Specify the methods used to assess risk of bias in the included studies, including details of the tool(s) used, how many reviewers assessed each study and whether they worked independently, and if applicable, details of automation tools used in the process.	Section 2.5; Supplementary Methods S6, S11 and S15–S19; Supplementary Appraisal S3.1; Supplementary Assessment Details. Original assessments underwent human verification; additional and changed revision materials underwent independent review by Sang Jun Byun and final decisions by Byungwook Choi after AI-assisted review. Independent duplicate appraisal and complete source-level verification of every PROBAST+AI item are not claimed.
Effect measures	12	Specify for each outcome the effect measure(s) (e.g. risk ratio, mean difference) used in the synthesis or presentation of results.	Section 2.6; Sections 3.2–3.5 and Table 2. HRs, discrimination measures and other reported prognostic metrics are distinguished.
Synthesis methods	13a	Describe the processes used to decide which studies were eligible for each synthesis (e.g. tabulating the study intervention characteristics and comparing against the planned groups for each synthesis (item #5)).	Sections 2.2 and 2.6; Supplementary Methods S5 and S7.
	13b	Describe any methods required to prepare the data for presentation or synthesis, such as handling of missing summary statistics, or data conversions.	Sections 2.4 and 2.6; Supplementary Methods S7. Unverified predictor scales are flagged and missing uncertainty is not imputed.
	13c	Describe any methods used to tabulate or visually display results of individual studies and syntheses.	Section 3.1; Tables 1–3; Supplementary Methods S5 and S8; Supplementary Data.
	13d	Describe any methods used to synthesize results and provide a rationale for the choice(s). If meta-analysis was performed, describe the model(s), method(s) to identify the presence and extent of statistical heterogeneity, and software package(s) used.	Section 2.6; Supplementary Methods S6, Evidence synthesis and certainty. Structured narrative synthesis; no meta-analysis.
	13e	Describe any methods used to explore possible causes of heterogeneity among study results (e.g. subgroup analysis, meta-regression).	Section 2.6 describes stratified narrative interpretation. No statistical subgroup analysis or meta-regression was performed.

PRISMA

PRISMA 2020 Checklist

Section and Topic	Item #	Checklist item	Location where item is reported
	13f	Describe any sensitivity analyses conducted to assess robustness of the synthesized results.	Section 2.6; Supplementary Methods S18. Descriptive sensitivity synthesis restricted the evidence to Core reports, then to aligned comparisons, separating multivariable-model AUC comparisons. No pooled-effect sensitivity analysis was performed.
Reporting bias assessment	14	Describe any methods used to assess risk of bias due to missing results in a synthesis (arising from reporting biases).	No formal synthesis-level assessment of bias due to missing results is reported. Study-level selective reporting is considered in Section 2.5 and Supplementary Appraisal.
Certainty assessment	15	Describe any methods used to assess certainty (or confidence) in the body of evidence for an outcome.	Sections 2.5–2.6; Supplementary Methods S6. No formal certainty-of-evidence grading was performed. Disease- and outcome-specific confidence statements are qualitative interpretations, not formal certainty grades.
**RESULTS**			
Study selection	16a	Describe the results of the search and selection process, from the number of records identified in the search to the number of studies included in the review, ideally using a flow diagram.	Section 3.1; Figure 1; Supplementary Methods S23–S24. Original database branch: 101 assessed, 69 excluded, 32 included. Two Google Scholar reports and one revision inclusion yield 35 reports. Revision branch: 2402 exported, 18 duplicate links, 2384 active, 2383 excluded and one included. Revision exclusions are not added to the original 69.
	16b	Cite studies that might appear to meet the inclusion criteria, but which were excluded, and explain why they were excluded.	Section 3.1; Supplementary Excluded Reports.csv lists 69 original-branch full-text exclusions and reasons. Supplementary Methods S24–S25 describes selected revision exclusions and source-access limitations. The revision branch is accounted for separately.
Study characteristics	17	Cite each included study and present its characteristics.	Sections 3.1–3.5; Tables 1–2; Supplementary Data report catalogue and cohort records.
Risk of bias in studies	18	Present assessments of risk of bias for each included study.	Section 3.6; Supplementary Appraisal Tables S3.1–S3.3; Supplementary Assessment Details; Supplementary Data. QUIPS covers 62 scopes from 33 reports, Form S covers 6 scopes from 5 reports, and PROBAST+AI covers 119 records from 10 reports. Appraisal records and scopes are not independent studies.
Results of individual studies	19	For all outcomes, present, for each study: (a) summary statistics for each group (where appropriate) and (b) an effect estimate and its precision (e.g. confidence/credible interval), ideally using structured tables or plots.	Sections 3.2–3.5; Table 2; Supplementary Data effect records. Unreported CIs and unresolved predictor scales are identified rather than imputed.
Results of syntheses	20a	For each synthesis, briefly summarise the characteristics and risk of bias among contributing studies.	Sections 3.2–3.6; Tables 1–3; Supplementary Appraisal and Supplementary Data.
	20b	Present results of all statistical syntheses conducted. If meta-analysis was done, present for each the summary estimate and its precision (e.g. confidence/credible interval) and measures of statistical heterogeneity. If comparing groups, describe the direction of the effect.	No pooled statistical synthesis was performed. Narrative findings are presented in Sections 3.2–3.6; the rationale for not pooling is in Sections 2.6 and 3.6.

PRISMA 2020 Checklist

Section and Topic	Item #	Checklist item	Location where item is reported
	20c	Present results of all investigations of possible causes of heterogeneity among study results.	Narrative differences are discussed in Sections 3.2–3.6 and 4.2–4.5. No statistical heterogeneity investigation was performed.
	20d	Present results of all sensitivity analyses conducted to assess the robustness of the synthesized results.	Section 3.6; Supplementary Methods S18. Core restriction retained 13 reports; 23 aligned comparisons from 8 reports comprised 16 HR, 6 AUC and 1 concordance comparison. Separating 4 multivariable-model AUC comparisons left 19 burden comparisons. These descriptive restrictions did not establish general equivalence or superiority and did not support pooling.
Reporting biases	21	Present assessments of risk of bias due to missing results (arising from reporting biases) for each synthesis assessed.	No formal synthesis-level reporting-bias assessment is presented. Study-level reporting concerns are described in Section 3.6 and Supplementary Appraisal; review limitations are in Section 4.6.
Certainty of evidence	22	Present assessments of certainty (or confidence) in the body of evidence for each outcome assessed.	Not assessed formally; see Sections 2.5–2.6 and Supplementary Methods S6. Limitations affecting confidence are discussed in Sections 3.6, 4.1 and 4.6 and qualified in the Abstract and Conclusions.
**DISCUSSION**			
Discussion	23a	Provide a general interpretation of the results in the context of other evidence.	Sections 4.1–4.5.
	23b	Discuss any limitations of the evidence included in the review.	Sections 4.2–4.6.
	23c	Discuss any limitations of the review processes used.	Section 4.6; Supplementary Methods S4, S6 and S24–S25. Limitations include unavailable database subscriptions from study inception, incomplete recovery of technical, multidisciplinary and conference literature through supplementary searching, source-access gaps and absence of independent duplicate extraction and appraisal from scratch. Additional and changed revision materials were independently reviewed by Sang Jun Byun before final decisions by Byungwook Choi.
	23d	Discuss implications of the results for practice, policy, and future research.	Section 4.7 and Section 5.
**OTHER INFORMATION**			
Registration and protocol	24a	Provide registration information for the review, including register name and registration number, or state that the review was not registered.	Section 2.1: PROSPERO CRD420261497171; registered 6 September 2026. Timing relative to screening is disclosed.
	24b	Indicate where the review protocol can be accessed, or state that a protocol was not prepared.	Section 2.1; Supplementary Methods S6. PROSPERO URL is supplied. A full internal protocol was prepared but has not been publicly released, as disclosed in the registration record.
	24c	Describe and explain any amendments to information provided at registration or in the protocol.	Section 2.1; Supplementary Methods S6, S11–S20 and S23–S25 describe implemented methods, revision additions, adopted appraisal changes, descriptive sensitivity restrictions and final disposition accounting. Historical stages are distinguished from current totals.

PRISMA 2020 Checklist

Section and Topic	Item #	Checklist item	Location where item is reported
Support	25	Describe sources of financial or non-financial support for the review, and the role of the funders or sponsors in the review.	Funding statement identifies the Clinical Research Grant of Daegu Catholic University Medical Center in 2026 (RD-26-0044) and states that the funder had no role in design, conduct, analysis, interpretation or publication decisions. AI assistance is disclosed in Acknowledgments.
Competing interests	26	Declare any competing interests of review authors.	Conflicts of Interest statement.
Availability of data, code and other materials	27	Report which of the following are publicly available and where they can be found: template data collection forms; data extracted from included studies; data used for all analyses; analytic code; any other materials used in the review.	Data Availability Statement; Supplementary Data (XLSX and JSON), Supplementary Assessment Details (XLSX and JSON), Supplementary Appraisal, Supplementary Methods, Supplementary Excluded Reports and this checklist. The full internal protocol has not been publicly released.

From: Page MJ, McKenzie JE, Bossuyt PM, Boutron I, Hoffmann TC, Mulrow CD, et al. The PRISMA 2020 statement: an updated guideline for reporting systematic reviews. BMJ 2021;372:n71. doi: 10.1136/bmj.n71 [11 in the main manuscript]. This work is licensed under CC BY 4.0. To view a copy of this license, visit https://creativecommons.org/licenses/by/4.0/
