# Supplementary material for: Prognostic Evidence for Artificial Intelligence-Derived PET Tumour-Burden Biomarkers in Cancer: A Systematic Review
Source: Diagnostics (Basel). 2026 Oct 29;16(21):3564. doi: 10.3390/diagnostics16213564 (PMC13762039; doi:10.3390/diagnostics16213564)
Supplement: Supplementary file 1 [file diagnostics-16-03564-s001.zip › Supplementary Appraisal.pdf]

Supplementary Appraisal

QUIPS and Form S assessments and restrictions on the use of effect estimates

Report and effect identifiers correspond to Supplementary Data. Supplementary Methods Table S3 links report identifiers to the main manuscript reference numbers. Bracketed reference numbers below refer to that same reference list.

S3.1 Scope and interpretation

This supplement presents finalized QUIPS assessments [14] of 62 units from 33 reports, comprising 372 domain judgments, and Form S assessments of six selected segmentation evaluation scopes from five reports, comprising 48 domain judgments. Each QUIPS unit identifies an outcome, cohort and analysis context. Units may share patients and do not represent independent studies.These assessments do not provide overall study scores, rankings or certainty-of-evidence ratings. They apply to the reports identified within the documented search coverage and do not resolve the possibility of missed studies. Human verification did not constitute fully independent duplicate extraction or appraisal, and no formal certainty-of-evidence grading was performed.

The initial consistency review covered 312 QUIPS and Form S domain judgments and revised 25 records, including 11 rating changes. Source passages were checked for identified discrepancies; every domain was not reassessed against the full text. Hae Won Kim verified the original assessments before submission, and Byungwook Choi finalized the judgments. During revision, additional and changed appraisal materials underwent independent review by Sang Jun Byun following AI-assisted review, with final decisions by Byungwook Choi. Choi adopted 18 additional QUIPS scopes: Liang (2), Gutsche (2), Küper (4), six additional factor scopes (6), Jemaa full-cohort analyses (2) and Le Goff (2). These additions account for 108 domain judgments. Human verification and author adoption do not constitute independent duplicate assessment.

Clinical prediction model assessments comprise 119 records across ten reports: 39 development and 80 evaluation records, with 2064 item responses, 476 domain judgments and 357 applicability explanations. Development quality was High in 17 and Unclear in 22 records; evaluation risk of bias was High in 67 and Unclear in 13. Overall applicability concern was High in eight and Unclear in 111 records. The complete official PROBAST+AI form [15] was checked for structural correspondence; source-level validity of every response has not been verified. These records are not independent models or cohorts.

Confounding judgments concern whether an association can be interpreted as independent prognostic information. A high risk-of-bias judgment for an unadjusted hazard ratio does not negate the reported crude association, but limits its interpretation as an independent association. Adjustment in a separate prediction model does not make that hazard ratio adjusted. Form S was developed for this review to assess segmentation agreement and is not a validated instrument. It was applied to six selected evaluation scopes, not all included reports. Insufficient reporting was rated as unclear unless the available evidence supported a high risk-of-bias judgment.

Table S3.1 Domain distributions

QUIPS

Domain	Units	Low	Moderate	High	Unclear
Participation	62	3	45	14	0
Attrition	62	0	58	4	0
Factor measurement	62	2	60	0	0
Outcome measurement	62	39	23	0	0
Confounding	62	0	25	37	0
Analysis and reporting	62	0	12	50	0

Form S

Domain	Units	Low	Moderate	High	Unclear
Case selection	6	0	0	1	5
AI index method	6	5	0	0	1

Domain	Units	Low	Moderate	High	Unclear
Expert reference	6	1	0	1	4
Evaluation independence	6	4	0	1	1
Failures and missing data	6	0	0	0	6
Analysis unit and clustering	6	6	0	0	0
Agreement metric	6	2	0	0	4
Selective reporting	6	0	0	0	6

Counts refer to assessment units or evaluation scopes, not independent reports or patient populations. QUIPS judgments use low, moderate and high categories. Form S judgments use low, high and unclear categories; moderate is not an available response. Unclear indicates insufficient information for a judgment and should not be interpreted as low risk.

Table S3.2 QUIPS judgments by assessment unit

D1, study participation; D2, study attrition; D3, prognostic factor measurement; D4, outcome measurement; D5, study confounding; D6, statistical analysis and reporting. L, low; M, moderate; H, high. OS denotes overall survival; PFS, progression-free survival; and FFR, freedom from recurrence.
Report (R), outcome (O) and cohort (C) identifiers correspond to records in Supplementary Data. Analysis contexts distinguish assessments within the same cohort, including different measurement time points. Each row represents an assessment unit, and multiple rows may include the same patients.

Report	Outcome/cohort/analysis context	Outcome	D1	D2	D3	D4	D5	D6
R001	C001; baseline	OS	M	M	M	L	M	H
R001	C001; after 2 cycles	OS	M	M	M	L	M	H
R001	C001; after 4 cycles	OS	M	M	M	L	M	H
R002	C002; reported cohort	PFS	M	M	M	L	M	H
R002	C002; reported cohort	OS	M	M	M	L	M	H
R003	C003; reported cohort	PFS	M	M	M	L	H	H
R004	C004; reported cohort	OS	M	M	M	L	H	H
R006	C006; internal R-CHOP	PFS	M	M	M	M	M	H
R006	C006; external R-CHOP	PFS	M	M	M	M	M	M
R006	C006; internal R-CHOP	OS	M	M	M	L	M	H
R006	C006; external R-CHOP	OS	M	M	M	L	M	M
R007	C007; reported cohort	PFS	M	H	M	M	M	H
R008	C008; reported cohort	OS	L	M	M	L	M	H
R009	C009; reported cohort	OS	M	M	M	L	M	H
R010	C010; baseline	OS	M	M	M	L	H	H
R010	C010; 3 months	OS	M	M	M	L	H	H
R011	C011; reported cohort	Locoregional control	M	H	M	M	H	H
R012	C012; reported cohort	PFS	M	M	M	M	M	H
R012	C012; reported cohort	OS	M	M	M	M	M	H
R013	C013A; reported cohort	Freedom from distant metastases	M	M	M	L	H	H
R013	C013B; reported cohort	Freedom from distant metastases	M	M	M	L	H	M
R014	C014; reported cohort	PFS	M	M	M	M	M	H

Report	Outcome/cohort/analysis context	Outcome	D1	D2	D3	D4	D5	D6
R015	C015; reported cohort	PFS	M	M	M	L	M	H
R015	C015; reported cohort	OS	M	M	M	L	M	H
R016	C016A; reported cohort	OS	M	M	M	L	H	H
R016	C016A; reported cohort	PFS	M	M	M	M	H	H
R017	C017; reported cohort	OS	M	M	M	L	H	M
R018	C018A; reported cohort	OS	M	H	M	L	H	M
R018	C018B; reported cohort	OS	M	H	M	L	H	M
R019	C019; reported cohort	OS	M	M	M	L	H	M
R021	C021A; reported cohort	PFS	H	M	M	L	H	H
R021	C021A; reported cohort	OS	H	M	M	L	H	H
R021	C021B; reported cohort	PFS	H	M	M	L	H	H
R021	C021B; reported cohort	OS	H	M	M	L	H	H
R022	C022; reported cohort	FFR	M	M	M	L	H	H
R022	C022; reported cohort	OS	M	M	M	L	H	H
R023	C023B; reported cohort	PFS	M	M	M	L	M	H
R023	C023B; reported cohort	OS	M	M	M	L	M	H
R024	C024; reported cohort	OS	L	M	M	L	H	M
R025	C025; reported cohort	Prostate cancer-specific survival	H	M	M	L	M	H
R026	C026; reported cohort	OS	L	M	M	L	M	H
R027	C027; reported cohort	PFS	M	M	M	L	H	H
R027	C027; reported cohort	OS	M	M	M	L	H	H
R028	C028; reported cohort	OS	H	M	M	L	H	H
R032	C032; pretreatment burden; internal survival test	OS	M	M	M	L	H	M
R032	C032; pretreatment burden; internal survival test	DFS	M	M	M	M	H	M
R033	AI-derived MTV percentage change <=0% versus >0%	OS	M	M	M	M	H	H
R033	AI-derived MTV percentage change <=0% versus >0%	PFS	M	M	M	M	H	H
R034	baseline_PSMAvol_OS	OS	H	M	M	M	H	H
R034	change_PSMAvol_OS	OS	H	M	M	M	H	H
R034	PSMA organ score_OS	OS	H	M	M	M	H	H
R034	RECIP1.0 PD vs non-PD_OS	OS	H	M	M	M	H	H
R020	aTMTV>Q3	PFS	H	M	M	M	H	H
R020	aTMTV>Q3	OS	H	M	M	L	H	H
R029	MTV continuous	OS	M	M	M	M	H	H
R029	TLA continuous	OS	M	M	M	M	H	H
R005	AI-PET-GTV log(cm3)	LRF	M	M	L	M	M	M
R005	AI-PET-GTV log(cm3)	DM	M	M	L	M	M	M
R020	Full cohort n=1268; median/Q3 linked contrasts	PFS	H	M	M	M	H	H
R020	Full cohort n=1268; median/Q3 linked contrasts	OS	H	M	M	L	H	H
R035	C035; Baseline TMTV–PFS	PFS	M	M	M	M	M	H
R035	C035; Combined ctDNA/TMTV categories–PFS	PFS	M	M	M	M	M	H

Table S3.3 Form S judgments by evaluation scope

The six evaluation scopes represent five reports. Internal and external agreement evaluations from R006 are assessed separately. Judgments apply to the specified evaluation scope and should not be extended to all analyses in the report. S1, case selection; S2, AI index method; S3, expert reference; S4, evaluation independence; S5, failures and missing data; S6, analysis unit and clustering; S7, agreement metric; S8, selective reporting. L, low; H, high; U, unclear. ICC denotes intraclass correlation coefficient.

Report	Evaluation scope	S1	S2	S3	S4	S5	S6	S7	S8
R003	Paired agreement evaluation	H	U	H	U	U	L	L	U
R005	Paired agreement evaluation	U	L	L	L	U	L	U	U
R006	Internal ICC evaluation	U	L	U	H	U	L	U	U
R016	Paired agreement evaluation	U	L	U	L	U	L	L	U
R006	External ICC n=273	U	L	U	L	U	L	U	U
R013	External paired subset n=65	U	L	U	L	U	L	U	U

For R003, the supplementary material reports 1,192 scans, including 485 PET-negative scans. The available evidence does not establish that all PET-negative scans were omitted from the reported results. The prediction pathway used for evaluation and the completeness of failure reporting remain unclear.
For R006, the external ICC evaluation includes 273 patients. This agreement sample is distinct from the outcome-specific survival cohorts and should not be used as their sample size.
For R013, the external paired evaluation of the convolutional neural network includes 65 patients. The exploratory cohort assessed using adaptive thresholding and manual correction is not included as evidence of agreement for the network.

S3.4 Changes affecting interpretation

Reports	Changes and implications
R013, R016, R021, R022	Ten QUIPS confounding judgments changed from moderate to high because the retained hazard ratios came from univariable analyses. Adjustment in separate multivariable models does not establish that these hazard ratios were adjusted.
R006	Outcome measurement was assessed separately for OS and PFS. Death is explicitly included in the reported OS definition but is not explicitly mentioned in the PFS definition. This reporting gap does not establish that deaths were excluded from the PFS analysis.
R016	The assessed prognostic factor associations concern the 69-patient development cohort, not the separate 31-patient prediction model validation cohort. The PFS outcome measurement judgment changed from low to moderate because the endpoint definition and assessment procedures were insufficiently clear.
R013	The exploratory measurements used adaptive thresholding with manual review or correction. Use of the convolutional neural network in another evaluation does not establish its use in this cohort. Observer blinding must also be assessed separately for each evaluation scope.

Reports	Changes and implications
R003	Patient-level cross-validation was described, but it could not be verified that each evaluated prediction was generated by a model trained without that patient’s data.
R023	The report describes a 117-patient clinical evaluation and a receiver operating characteristic–derived cutoff of 201.2 cm^3. It remains unclear whether this prognostic cutoff was fixed before evaluation. This uncertainty does not establish that the cutoff was retuned in the external cohort.

Table S3.4 Hazard ratios with unresolved predictor scales

The 16 hazard ratios below were extracted from Table 3 of Girum et al. (R021) [23]. Their predictor coding or transformation could not be established from the available sources. The extracted estimates and 95% confidence limits are retained unchanged. These hazard ratios may be described with an explicit qualification that their predictor scales remain unresolved. They should not be pooled or directly compared in magnitude with other hazard ratios until their scales are verified. This restriction concerns these hazard ratios; separately reported AUCs remain available for descriptive comparison within their respective outcomes and evaluation settings.
No meta-analysis was performed in this review. The effect identifiers link these records to Supplementary Data.

Effect ID	HR	95% CI lower	95% CI upper
E195	11.24	2.1	46.2
E197	9	2.53	23.63
E199	11.81	3.29	31.77
E201	12.49	3.42	34.5
E203	16.43	2.42	77.29
E205	8.6	1.47	28.33
E207	22.14	4.73	69.06
E209	22.79	3.8	79.21
E211	13.79	0.45	86.8
E213	32.83	0.4	220.8
E215	9.24	0.95	37.94
E217	16.79	0.69	86.41
E219	64.3	0.74	384.8
E221	49.21	0.07	258.3
E223	14.17	0.59	67.02
E225	20.39	0.08	93.66

S3.5 Relationship to other supplementary files

Supplementary Methods describes the assessment methods and review-process limitations. Supplementary Data.xlsx and its JSON companion contain current extraction and model-assessment tables. Detailed QUIPS and Form S judgments, supporting rationales, source locations and recorded review provenance are supplied in Supplementary Assessment Details.xlsx and its JSON companion. The assessment JSON preserves the original history; historical draft or pending descriptions do not override the current adopted tables. Restrictions on individual effect estimates supplement extraction records without changing reported numerical values. Human verification and author adoption do not imply independent duplicate assessment. PROBAST+AI structural correspondence was checked, but source-level validity of every item remains unverified; see Supplementary Methods S11 and S19.
