# Supplementary material for: Prognostic Evidence for Artificial Intelligence-Derived PET Tumour-Burden Biomarkers in Cancer: A Systematic Review
Source: Diagnostics (Basel). 2026 Oct 29;16(21):3564. doi: 10.3390/diagnostics16213564 (PMC13762039; doi:10.3390/diagnostics16213564)
Supplement: Supplementary file 1 [file diagnostics-16-03564-s001.zip › Supplementary Methods.pdf]

**Supplementary Methods**

This supplement describes the search strategies, study selection, comparison definitions and differences between the planned and implemented review methods. Study-level data and cohort links are provided in Supplementary Data, comprising an Excel workbook and a JSON companion. Risk-of-bias assessments are presented separately in Supplementary Appraisal. Section S9 provides the 23 aligned comparisons and the basis for the restricted pooling assessment.

Reference numbers correspond to the main manuscript reference list and are not renumbered within this supplement.

**S1 Search execution and accounting**

PubMed and Embase.com were searched on 29 August 2026 without date or language restrictions in the search queries. Eligibility was restricted to English-language reports during screening. The searches combined concepts for positron emission tomography (PET), artificial intelligence (AI), segmentation or tumour burden, and cancer. Prognostic terms were not required.

PubMed returned 1786 records. The initial Embase search returned 5371 results, of which 2274 (42.3%) were classified as conference abstracts, conference papers or conference reviews. These publication types were excluded because their proportion exceeded the prespecified 30% threshold. Of the remaining 3097 results, 57 trial-registry records were set aside, leaving 3040 bibliographic records for screening. Thus, the conference-publication filter was applied before export rather than during screening, as described in earlier planning documents.

The original database searches identified 4826 records, comprising 1786 from PubMed and 3040 from Embase. After removal of 1638 duplicates, 3188 records underwent screening. In the reconciled original database branch, 3081 records were excluded and 107 records were considered for full-text retrieval. Removal of one additional duplicate left 106 unique reports sought for retrieval. Five reports could not be retrieved. Of the 101 reports assessed in this branch, 69 were excluded and 32 were included. Two additional Contextual reports were identified through Google Scholar. After re-inclusion of Le Goff et al. [26] from the original title/abstract exclusions, Küper et al. [20] were adopted as Contextual evidence from the separate revision-stage follow-up set, bringing the current catalogue to 35 reports from 31 catalogue families, comprising 13 Core and 22 Contextual reports. Figure 1 and Supplementary Methods S24 present the original and revision branches separately, preserving their different assessment levels. Liang et al. [34] were included on reassessment of the original database results. Gutsche et al. [19] were transferred from the previous E2 exclusion category to Core inclusion; that reclassification did not add an assessed report. Wang et al. [21] remained Contextual after correction of the comparator attribution.

**S2 Executed PubMed strategy**

(

"Positron-Emission Tomography"[MeSH Terms] OR

"Positron Emission Tomography Computed Tomography"[MeSH Terms] OR

"PET"[tiab] OR "PET/CT"[tiab] OR "PET-CT"[tiab] OR

"positron emission"[tiab] OR "FDG"[tiab] OR "fluorodeoxyglucose"[tiab] OR

"PSMA"[tiab] OR "DOTATATE"[tiab] OR "DOTATOC"[tiab]

)

AND

(

"Artificial Intelligence"[MeSH Terms] OR "Deep Learning"[MeSH Terms] OR

"Machine Learning"[MeSH Terms] OR "Neural Networks, Computer"[MeSH Terms] OR

"artificial intelligence"[tiab] OR "deep learning"[tiab] OR "machine learning"[tiab] OR

"neural network"[tiab] OR "neural networks"[tiab] OR "convolutional"[tiab] OR

"U-Net"[tiab] OR "nnU-Net"[tiab] OR "transformer"[tiab] OR

"radiomics"[tiab] OR "radiomic"[tiab] OR

"automated"[tiab] OR "automatic"[tiab] OR "fully automatic"[tiab] OR

"segment anything"[tiab] OR "foundation model"[tiab] OR "foundation models"[tiab] OR

"self-supervised"[tiab] OR "vision transformer"[tiab] OR "nnUNet"[tiab] OR

"deep-learning"[tiab] OR "artificial neural network"[tiab]

)

AND

(

"segmentation"[tiab] OR "segmented"[tiab] OR "segmenting"[tiab] OR

"delineation"[tiab] OR "delineated"[tiab] OR "contouring"[tiab] OR

"metabolic tumor volume"[tiab] OR "metabolic tumour volume"[tiab] OR

"MTV"[tiab] OR "TMTV"[tiab] OR

"total lesion glycolysis"[tiab] OR "TLG"[tiab] OR

"tumor burden"[tiab] OR "tumour burden"[tiab] OR

"tumor volume"[tiab] OR "tumour volume"[tiab] OR

"total tumor volume"[tiab] OR "total tumour volume"[tiab]

)

AND

(

"Neoplasms"[MeSH Terms] OR

"cancer"[tiab] OR "cancers"[tiab] OR "carcinoma"[tiab] OR "carcinomas"[tiab] OR

"tumor"[tiab] OR "tumors"[tiab] OR "tumour"[tiab] OR "tumours"[tiab] OR

"lymphoma"[tiab] OR "lymphomas"[tiab] OR "myeloma"[tiab] OR "sarcoma"[tiab] OR

"malignant"[tiab] OR "malignancy"[tiab] OR "neoplasm"[tiab] OR "neoplasms"[tiab]

)

**S3 Executed Embase strategy**

(('positron emission tomography'/exp OR 'positron emission tomography' OR 'positron emission tomography-computed tomography'/exp OR 'positron emission tomography-computed tomography' OR pet:ti,ab,kw OR 'pet ct':ti,ab,kw OR 'pet/ct':ti,ab,kw OR 'pet-ct':ti,ab,kw OR fdg:ti,ab,kw OR fluorodeoxyglucose:ti,ab,kw OR 'positron emission':ti,ab,kw OR psma:ti,ab,kw OR dotatate:ti,ab,kw OR dotatoc:ti,ab,kw) AND ('artificial intelligence'/exp OR 'deep learning'/exp OR 'machine learning'/exp OR 'artificial neural network'/exp OR 'convolutional neural network'/exp OR 'artificial intelligence':ti,ab,kw OR 'deep learning':ti,ab,kw OR 'machine learning':ti,ab,kw OR 'neural network*':ti,ab,kw OR convolutional:ti,ab,kw OR 'u-net':ti,ab,kw OR 'nnu-net':ti,ab,kw OR 'nnunet':ti,ab,kw OR transformer:ti,ab,kw OR 'vision transformer':ti,ab,kw OR 'segment anything':ti,ab,kw OR 'foundation model*':ti,ab,kw OR 'self-supervised':ti,ab,kw OR radiomic*:ti,ab,kw OR automated:ti,ab,kw OR automatic:ti,ab,kw) AND ('tumor volume'/exp OR segmentation:ti,ab,kw OR segmented:ti,ab,kw OR segmenting:ti,ab,kw OR delineation:ti,ab,kw OR delineated:ti,ab,kw OR contouring:ti,ab,kw OR 'metabolic tumor volume':ti,ab,kw OR 'metabolic tumour volume':ti,ab,kw OR mtv:ti,ab,kw OR tmtv:ti,ab,kw OR 'total lesion glycolysis':ti,ab,kw OR tlg:ti,ab,kw OR 'tumor burden':ti,ab,kw OR 'tumour burden':ti,ab,kw OR 'tumor volume':ti,ab,kw OR 'tumour volume':ti,ab,kw OR 'total tumor volume':ti,ab,kw OR 'total tumour volume':ti,ab,kw) AND ('neoplasm'/exp OR cancer*:ti,ab,kw OR tumor*:ti,ab,kw OR tumour*:ti,ab,kw OR carcinoma*:ti,ab,kw OR lymphoma*:ti,ab,kw OR myeloma*:ti,ab,kw OR sarcoma*:ti,ab,kw OR malignan*:ti,ab,kw OR neoplas*:ti,ab,kw)) NOT ((('positron emission tomography'/exp OR 'positron emission tomography' OR 'positron emission tomography-computed tomography'/exp OR 'positron emission tomography-computed tomography' OR pet:ti,ab,kw OR 'pet ct':ti,ab,kw OR

'pet/ct':ti,ab,kw OR 'pet-ct':ti,ab,kw OR fdg:ti,ab,kw OR fluorodeoxyglucose:ti,ab,kw OR 'positron emission':ti,ab,kw OR psma:ti,ab,kw OR dotatate:ti,ab,kw OR dotatoc:ti,ab,kw) AND ('artificial intelligence'/exp OR 'deep learning'/exp OR 'machine learning'/exp OR 'artificial neural network'/exp OR 'convolutional neural network'/exp OR 'artificial intelligence':ti,ab,kw OR 'deep learning':ti,ab,kw OR 'machine learning':ti,ab,kw OR 'neural network*':ti,ab,kw OR convolutional:ti,ab,kw OR 'u-net':ti,ab,kw OR 'nnu-net':ti,ab,kw OR 'nnunet':ti,ab,kw OR transformer:ti,ab,kw OR 'vision transformer':ti,ab,kw OR 'segment anything':ti,ab,kw OR 'foundation model*':ti,ab,kw OR 'self-supervised':ti,ab,kw OR radiomic*:ti,ab,kw OR automated:ti,ab,kw OR automatic:ti,ab,kw) AND ('tumor volume'/exp OR segmentation:ti,ab,kw OR segmented:ti,ab,kw OR segmenting:ti,ab,kw OR delineation:ti,ab,kw OR delineated:ti,ab,kw OR contouring:ti,ab,kw OR 'metabolic tumor volume':ti,ab,kw OR 'metabolic tumour volume':ti,ab,kw OR mtv:ti,ab,kw OR tmtv:ti,ab,kw OR 'total lesion glycolysis':ti,ab,kw OR tlg:ti,ab,kw OR 'tumor burden':ti,ab,kw OR 'tumour burden':ti,ab,kw OR 'tumor volume':ti,ab,kw OR 'tumour volume':ti,ab,kw OR 'total tumor volume':ti,ab,kw OR 'total tumour volume':ti,ab,kw) AND ('neoplasm'/exp OR cancer*:ti,ab,kw OR tumor*:ti,ab,kw OR tumour*:ti,ab,kw OR carcinoma*:ti,ab,kw OR lymphoma*:ti,ab,kw OR myeloma*:ti,ab,kw OR sarcoma*:ti,ab,kw OR malignan*:ti,ab,kw OR neoplas*:ti,ab,kw)) AND ([conference abstract]/lim OR [conference paper]/lim OR [conference review]/lim))

The Embase.com search used preferred-term mapping, free-text searching, narrower-term explosion and broad searching. Major-focus restriction was not applied. The search string reproduced above is the saved query used for export and includes the conference-publication exclusion.

### S4 Supplementary searches and exclusion accounting

The supplementary-search record was finalized on 9 September 2026. The first 200 ranked Google Scholar results were examined. Of these, 108 represented previously identified reports or duplicate entries, leaving 92 additional records for screening. Ninety were excluded, and two were included as Contextual reports. The available closure record did not document the exact executed query or the account and personalization settings. These gaps limit the reproducibility of the Google Scholar search.

Backward citation searching examined 988 citation instances across the original 31 included reports, including all 19 references of Luo et al. (2026) [25]. These were not deduplicated unique records and were not added to the database or Google Scholar record totals. No additional eligible report absent from the database results was identified. Leung et al. (2024) [30], which had already been identified through the database searches, was reassessed and included after verification of its AI-derived tumour burden measurement and prognostic analysis. Forward citation searching was performed on 29 August 2026 using the PubMed cited-by interface (NCBI E-utilities, pubmed_pubmed_citedin) for 10 seed reports. It

identified 159 unique citing records. Of these, 51 were captured by the initial PubMed strategy and another six were present in the finalized PubMed/Embase union, leaving 102 records for separate screening. Choi and Byun independently assessed these 102 records and both excluded all of them. Four disagreements in exclusion-reason coding were resolved following review by Kim. No additional eligible report was identified. These 102 records were assessed separately from the 3188 database records and were not added to the database-screening denominator. The citation search also informed vocabulary expansion of the final PubMed strategy. Its coverage was limited to PubMed citation links.

| Code | Excluded reports | Definition |
|---|---|---|
| E1 | 4 | Segmentation used no learning component, such as a purely threshold-based method. These methods remained eligible as comparators. |
| E2 | 6 | No survival or prognostic result was reported for the AI-derived tumour burden measurement. This included studies reporting segmentation accuracy alone or prognostic analyses limited to other parameters. |
| E4 | 14 | Ineligible publication type, including reviews, editorials, case reports, conference abstracts alone and letters. The exception for the proceedings report by Luo et al. (2026) is described below. |
| E8 | 3 | Preprint without an identifiable peer-reviewed publication. |
| E9 | 36 | An AI prognostic or predictive model used PET images but did not produce an eligible metabolic tumour burden measurement, or did not report that measurement as a study variable. |
| E10 | 4 | The imaging modality or population was outside the scope of PET or PET/CT studies in patients with malignancy. |
| E11 | 2 | Other PET or PET/CT studies in patients with malignancy that did not produce an eligible AI-derived metabolic tumour burden measurement. These included diagnostic, staging, detection, classification or segmentation studies without a prognostic model, and studies reporting only non-burden measures such as tumour dissemination. |

For preprints, the eligibility procedure required a search for a peer-reviewed publication before exclusion, with the date, search route and assessor recorded. When both versions were retrieved, they were treated as linked reports rather than duplicate records.

E1 took precedence over E11 when an otherwise eligible tumour burden measurement was obtained without a learning-based step. E11 covered studies without an eligible AI-derived tumour burden measurement that were not classified under an earlier exclusion criterion.

The exclusion counts refer to 69 unique reports. Earlier records contained 71 entries; deduplication reduced these to 70 unique reports, and reclassification of PM00774 reduced the current exclusions to 69. No reports were excluded under criteria E3, E5 or E7. Supplementary Excluded Reports provides the title, DOI and exclusion reason for the excluded reports; the PM00774 amendment removes this report from the historical list. The five reports not retrieved are outside this list. PM00263 and PM00665 are included reports following documented reassessment and are not listed as exclusions. EM00065 and EM00542 represent the same DOI and are counted once.

Conference abstracts alone were listed under the publication-type exclusion criterion. However, the proceedings report by Luo et al. (2026) [25], identified through supplementary searching, was retained as Contextual evidence and assessed only from its abstract. The complete proceedings text was not available for assessment. This exception created a difference in the availability of evidence across search routes and limited assessment of the report's methods and findings. The revision audit included conference records, but all 52 recorded conference dispositions retained E4. Journal companions were considered separately where available. This did not constitute a comprehensive conference-literature search or a sensitivity synthesis including conference abstracts and cannot establish absence of publication-type bias.

During revision, temporal eligibility was checked using the first online publication date while retaining 9 September 2026 as the original temporal eligibility boundary. The 13 October revision export did not extend this boundary or constitute a comprehensive update through the revision date. Liang et al. (doi:10.1038/s41698-026-01283-7 [34]; first online 29 January 2026) were included as Contextual evidence following reassessment of their automated MTV/TLG survival analyses. A targeted assessment also considered the Emmett ASCO 2025 abstract (doi:10.1200/JCO.2025.43.16_suppl.5054 [44]), its related RANZCR poster (doi:10.26044/ranzcr2025/C-489 [45]), and the Tricarico 2024 abstract (doi:10.1016/j.mednuc.2024.01.005 [46]). These conference reports were documented separately from the main synthesis; the Emmett reports were treated as companions from the same cohort. This targeted assessment was not a comprehensive conference-literature search. Ferdinandus et al. (doi:10.1186/s40658-026-00935-8 [47]; first online 10 September 2026) and Zhang et al. (doi:10.3390/cancers18193196 [48]; first online 3 October 2026) fell after the 9 September search closure.

**S5 Comparison definitions and reconciliation**

Comparisons were classified according to the measurements, analysis populations and prognostic contrasts reported. The aligned burden category required a common analysis population and a compatible burden contrast or discrimination metric and contained 23 comparison records. This descriptive classification did not establish equivalence or suitability for pooling. Interpretation also depended on covariate adjustment, outcome and time horizon, predictor scale, threshold selection, patient overlap and uncertainty in the paired comparison. Comparisons involving two-dimensional burden areas, dissemination measures, different segmentation targets, different tracers, method-specific thresholds, unequal nested samples or gland-level measurements were classified separately. Tables S1 and S2 in Section S9 report the numerical results and comparison conditions, respectively.

Supplementary Data contains 69 comparison records, including a corrected record for Wang et al. (2025) [21]. In that study, the areas under the receiver operating characteristic curve (AUCs) of 0.61 and 0.59 represented AI-derived total metabolic tumour volume and total lesion glycolysis, respectively. They did not constitute an AI-versus-expert comparison. The record was retained with this correction and excluded from the AI-versus-expert synthesis; the report remained classified as Contextual evidence. Detailed comparison links and the historical extraction records are retained in Supplementary Data.

AUC values were retained on their reported scale, with any conversion between percentages and proportions requiring explicit documentation. For Kovacs et al. (2026) [27], the manual 1-year AUC was 77.0% in the source report's Table 2 and Results but 76.9% in its abstract. The review's comparison table used 77.0% from the source table. The discrepancy was documented without altering the historical extraction snapshot.

**S6 Appraisal and protocol reconciliation**

Registered methods: https://www.crd.york.ac.uk/PROSPERO/view/CRD420261497171. A full protocol was prepared as an internal working document but has not been publicly released. Differences between the planned and implemented methods are described below.

Dated protocol records distinguish pre-screening decisions from subsequent specifications. On 29 August 2026, D-16 documented the Core/Contextual criteria, D-18 prioritized paired comparisons, D-19 specified conditional quantitative synthesis with a narrative alternative, and D-23 addressed shared-cohort dependence. The appraisal framework was also documented before screening (D-20 and D-45). These records document planned procedures, not confirmation that every procedure was implemented. Formal title and abstract screening began on 2 September (D-105). PROSPERO submission occurred on 5 September and registration on 6 September (CRD420261497171), before data extraction began.

On 4 September, after screening began but before extraction, D-123 specified a structured outcome-specific certainty assessment based on the amount, directness, consistency, precision and risk of bias of the evidence, without formal GRADE categories. D-125 specified REML estimation and conditional HKSJ/Wald procedures for any eligible quantitative synthesis. These specifications are distinct from the pre-screening Core/Contextual criteria and conditional synthesis framework. No quantitative meta-analysis was performed, so those estimator specifications were not applied. No formal certainty grades were assigned; this should not be taken as evidence that the planned structured outcome-specific assessment was completed. Differences between planned and implemented procedures are described below.

**Prognostic factor associations**

The protocol specified the six Quality in Prognosis Studies (QUIPS) [14] domains. The finalized inventory comprises 62 assessment units from 33 reports. AI assisted assessment and consistency checking. Hae Won Kim verified the original judgments; Byungwook Choi finalized them after discussion. Choi adopted two Liang [34] units on 13 October 2026 and two Gutsche [19] and four Küper [20] units on 15 October 2026 after reviewing AI-assisted proposals and source-based rationales. These were researcher-verified assessments, not independent duplicate reviews. They supersede the historical review-specific grid, retained for documentation rather than treated as QUIPS. S10 maps report-level coverage and six additional scopes (Tables S3–S4), now included after separate researcher adoption on 15 October 2026.

**Clinical prediction models**

The protocol specified assessment of model development quality, risk of bias in model evaluation and applicability using PROBAST+AI [15]. The implemented assessment used a framework based on PROBAST+AI and comprised 119 model-assessment records and 476 domain judgments across 10 reports. The inventory contains 39 development and 80 evaluation records, with 624 and 1440 item responses, respectively. These counts represent assessment records rather than independent models or studies. The 476 domain judgments concern development quality or evaluation risk of bias; the separate applicability addendum contains three domain explanations per record, giving 357 explanations rather than 357 independent assessments.

Full-text articles were available for nine reports, whereas the proceedings report by Luo et al. (2026) [25] was assessed from its abstract. AI tools assisted with the assessments, and Byungwook Choi confirmed the recorded judgments on 13 September 2026. Hae Won Kim verified the model assessments against the available source material before submission; questions were checked and discussed with Choi, who made the final judgments. At initial submission, correspondence with the complete official PROBAST+AI form had not been verified. The revision structural check is described below; source-

level validity of every response remains unverified, and the abstract-only report retains substantial information limitations.

During revision, the 16 development and 18 evaluation item concepts were first mapped to Table 2 of the primary PROBAST+AI [15] publication and then checked against the complete official form and E&E Light. The expanded comparison identified conditional-item and rationale issues, summarized in S11; it does not establish complete implementation or validate every answer. An AI-assisted applicability addendum linked all 119 records to the review question through the population, predictor definition, outcome and intended prediction time. Baseline and updated prediction were distinguished, clinical-only comparators were evaluated within their comparison question, and combined-score performance was not attributed solely to tumour burden. Resampling, repeated outcomes and overlapping cohorts were not counted as independent evidence.

The original assessments and subsequent adopted changes are preserved in the audit trail. The researcher also adopted the review-level PICOTS clarification, applicability explanations and four Kovacs [27] development assessments on 15 October 2026. The resulting inventory comprises 119 records (39 development and 80 evaluation), 2064 item responses, 476 quality or risk-of-bias domain judgments and 357 applicability explanations. Development quality was High in 17 and Unclear in 22 records; evaluation risk of bias remained High in 67 and Unclear in 13. Overall applicability concern was High in eight and Unclear in 111 records. These are assessment records, not independent models or patient cohorts. Source provenance distinguishes primary material, archived extractions and abstract-only information. The adopted Unclear ratings retain uncertainty about applicability and source reporting; adoption does not establish complete source-level verification or independent duplicate assessment.

### Segmentation agreement

Segmentation agreement was assessed using Form S, an eight-domain form developed for this review. The assessment covered six segmentation evaluation scopes. AI tools assisted with assessment and consistency checking, and Hae Won Kim verified the judgments before submission. Byungwook Choi finalized them after discussion of questions. Form S is not a validated instrument. The finalized QUIPS and Form S judgments are summarized in Supplementary Appraisal. Domain-level rationales, source locations and reviewer confirmation records are provided in the accompanying Supplementary Assessment Details workbook and JSON file.

### Diagnostic accuracy

The protocol specified QUADAS-2 assessment if a diagnostic accuracy synthesis was undertaken. No such synthesis was performed, and QUADAS-2 was not applied.

**Reporting completeness**

The protocol identified CLAIM 2024 [16] and TRIPOD+AI [17] as relevant reporting guidelines. Reporting limitations were considered narratively. No formal checklist completion scores or percentages were calculated, and these considerations were kept separate from the risk-of-bias judgments.

**Review verification**

Byungwook Choi and Sang Jun Byun independently screened the titles and abstracts of all 3188 records and recorded their decisions separately; disagreements were reconciled before full-text assessment. Hae Won Kim, a nuclear medicine physician, verified the full-text eligibility decisions, extracted data and critical appraisals before submission. Sang Jun Byun checked all Core reports. When Kim raised questions, Choi and Kim checked the source material and discussed the issue; Choi made the final decision. AI tools assisted with extraction, assessment and consistency checking. These procedures involved verification of extracted data and judgments rather than independent duplicate extraction and appraisal. Following AI-assisted review, Sang Jun Byun independently reviewed the additional and changed revision materials, and Byungwook Choi made the final decisions. During revision, the new Liang [34] extraction and two QUIPS units were finalized separately by Choi on 13 October 2026. We sought to extract eligible prognostic results as comprehensively as possible from the available reports. Study investigators were not contacted to request additional data. All authors reviewed and revised the manuscript and approved its final content.

**Evidence synthesis and certainty**

The protocol envisaged quantitative synthesis where outcomes, predictor scales and cohorts were sufficiently compatible. The review used a structured narrative synthesis. Differences in populations, outcomes, predictor scales and thresholds precluded a pooled hazard ratio and corresponding confidence interval. Incomplete reporting of uncertainty in paired comparisons also prevented pooling of differences between AI-derived and expert-derived measurements. No formal certainty-of-evidence rating was assigned. Similar estimates or overlapping confidence intervals were not interpreted as evidence of equivalence or interchangeability. Confidence is discussed by disease and outcome using the amount and independence of evidence, directness of the measurement and comparator, consistency, precision, confounding and analysis risk, and validation setting. The synthesis separates unadjusted association from independent prognostic information, model performance from burden-factor effects, and segmentation testing from external prognostic validation. Outcome-selected thresholds, overlapping cohorts and missing paired uncertainty limit transportability and comparative claims.These are qualitative interpretations linked to the existing appraisals, not formal certainty grades or a completed formal outcome-

specific certainty assessment. Incomplete search coverage and the absence of fully independent duplicate extraction and appraisal further limit these interpretations. Core membership and aligned-comparison status do not imply high certainty of evidence.

**Historical assessment records**

The earlier review-specific assessment grid was retained in Supplementary Data to document the assessment history. Its overall labels were not treated as results from a validated risk-of-bias instrument or used to assign certainty of evidence. The finalized QUIPS and Form S assessments are presented in Supplementary Appraisal. Clinical prediction model assessments are recorded separately in Supplementary Data, with the source limitations described above. Structural correspondence with the complete official PROBAST+AI form was checked during revision, but source-level validity of every response remains unverified.

**S7 Cohort dependency and source hierarchy**

The review included 35 reports from 31 catalogue families. Supplementary Data contains 41 cohort records describing the datasets used in these reports. These records do not represent 41 independent patient populations, and their sample sizes cannot be summed to obtain a unique patient total.

Shared cohorts or source datasets were identified in reports using REMARC, the head-and-neck datasets that included TCIA Head-Neck-Radiomics-HN1/MAASTRO, and the registered Western Australian prostate cancer cohort. Exact participant overlap was not enumerated for all linked analyses, so estimates from reports within the same cohort family were not assumed to be independent. Shared software or model-development history alone was not considered evidence of patient overlap. Supplementary Data documents seven relationship records, distinguishing cohort or source-dataset links from software lineage and methodological connections.

Numerical values were checked against the original reports, with source locations recorded. Discrepancies between abstracts, main texts and tables were documented, together with the value selected for the synthesis and the reason for that selection. Missing measures of uncertainty were not imputed.

**S8 Supplementary data contents and interpretation**

Supplementary Data comprises an Excel workbook and a JSON companion. The workbook contains the report catalogue, comparative summaries, cohort records and links, extracted effect estimates, references and clinical prediction model assessments. The JSON companion also preserves the original extraction records, including model descriptions,

segmentation definitions, outcomes and extraction notes.

The original extraction snapshot is retained as a historical record and may contain classifications that were subsequently revised. The current report catalogue and confirmed counts take precedence for report classification and cohort-family totals. The review includes 35 reports from 31 catalogue families, comprising 13 Core and 22 Contextual reports. Corrections to comparison links and their interpretation are documented separately from the original effect estimates.

Supplementary Appraisal presents the finalized QUIPS and Form S assessments and restrictions on quantitative synthesis, superseding the earlier appraisal entries in Supplementary Data. Section S6 describes limitations of the review and clinical prediction model assessments.

**S9 Aligned comparisons and restricted pooling assessment**

The aligned set contains 23 comparison records from eight reports in seven catalogue cohort families: 16 hazard-ratio (HR), six area-under-the-curve (AUC) and one concordance comparison. All use FDG. Family counts are catalogue identifiers, not counts of independent outcome cohorts. Four Dirks [37] model-AUC comparisons are reported separately from burden-only evidence. Two Weber [33] organ-specific HR comparisons require qualification for method-specific stepwise adjustment. The tables preserve the current aligned set and do not add the four Jemaa [9] comparisons whose cutoff alignment and adjustment specification remain unresolved.

Each row compares AI and expert results within the same analysis population. Tables S1 and S2 are joined by the comparison identifier. Confidence intervals describe each estimate, not the AI-minus-expert difference. Missing intervals, correlations and paired standard errors were not imputed. The alignment criteria organize the synthesis and do not establish equivalence or suitability for pooling.

**Table S1 Numerical results for the 23 aligned comparisons**

| ID | Report and cohort | n | Predictor and outcome horizon | Measure | AI estimate (95% CI) | Expert estimate (95% CI) |
|---|---|---|---|---|---|---|
| P001 | Nikulin et al., 2026 [10] C003 / CF003 | 534 | TMTV; PFS Time-to-event; no fixed prediction horizon | HR | 3.7 (CI NR) | 3.5 (CI NR) |
| P020 | Zschaeck et al., 2025 [4] C013B / CF013 | 65 | TLG; FFDM Time-to-event; no fixed prediction horizon | HR | 3.1 (CI NR) | 3.47 (CI NR) |
| P021 | Dirks et al., 2023 [37] C016A / CF016 | 69 | TMTV; OS Time-to-event; no fixed prediction horizon | HR | 14.3 (5.72–35.8) | 12.2 (4.95–29.8) |
| P022 | Dirks et al., 2023 [37] C016A / CF016 | 69 | TMTV; PFS Time-to-event; no fixed prediction horizon | HR | 4.23 (2.04–8.77) | 3.85 (1.87–7.94) |
| P023 | Dirks et al., 2023 [37] C016A / CF016 | 69 | TLG; OS Time-to-event; no fixed prediction horizon | HR | 7.77 (3.62–16.7) | 7.77 (3.62–16.7) |
| P024 | Dirks et al., 2023 [37] C016A / CF016 | 69 | TLG; PFS Time-to-event; no fixed prediction horizon | HR | 3.43 (1.59–7.38) | 3.18 (1.48–6.82) |
| P025 | Dirks et al., 2023 [37] C016A / CF016 | 69* | TMTV; OS 1-year OS AUC | AUC | 0.78 (0.65–0.91) | 0.8 (0.67–0.91) |
| P026 | Dirks et al., 2023 [37] C016A / CF016 | 69* | TMTV; OS 2-year OS AUC | AUC | 0.7 (0.56–0.85) | 0.69 (0.54–0.83) |

| ID | Report and cohort | n | Predictor and outcome horizon | Measure | AI estimate (95% CI) | Expert estimate (95% CI) |
|---|---|---|---|---|---|---|
| P027 | Dirks et al., 2023 [37] C016A / CF016 | 69* | TMTV; PFS 1-year PFS AUC | AUC | 0.61 (0.48–0.76) | 0.59 (0.45–0.73) |
| P028 | Dirks et al., 2023 [37] C016A / CF016 | 69* | TMTV; PFS 2-year PFS AUC | AUC | 0.42 (0.25–0.6) | 0.4 (0.22–0.6) |
| P029 | Nikulin et al., 2023 [3] C018A / CF013 | 585 | TTB; OS Time-to-event; no fixed prediction horizon | HR | 1.8 (CI NR) | 1.9 (CI NR) |
| P030 | Nikulin et al., 2023 [3] C018B / CF013 | 142 | TTB; OS Time-to-event; no fixed prediction horizon | HR | 1.9 (CI NR) | 1.8 (CI NR) |
| P039 | Han et al., 2022 [32] C022 / CF020 | 114 | MTV; FFR Time-to-event; no fixed prediction horizon | HR | 2.11 (1.09–4.06) | 2.43 (1.33–4.45) |
| P040 | Han et al., 2022 [32] C022 / CF020 | 114 | TLG; FFR Time-to-event; no fixed prediction horizon | HR | 1.9 (1.12–3.23) | 2.24 (1.34–3.75) |
| P041 | Han et al., 2022 [32] C022 / CF020 | 114 | MTV; OS Time-to-event; no fixed prediction horizon | HR | 1.73 (0.63–4.77) | 1.71 (0.66–4.41) |
| P042 | Han et al., 2022 [32] C022 / CF020 | 114 | TLG; OS Time-to-event; no fixed prediction horizon | HR | 1.57 (0.7–3.5) | 1.56 (0.72–3.37) |
| P043 | Borrelli et al., 2022 [2] C024 / CF022 | 106 | TLG; OS Time-to-event; no fixed prediction horizon | HR | 1.64 (1.21–2.21) | 1.54 (1.14–2.07) |

| ID | Report and cohort | n | Predictor and outcome horizon | Measure | AI estimate (95% CI) | Expert estimate (95% CI) |
|---|---|---|---|---|---|---|
| P044 | Weber et al., 2021 [33] C026 / CF024 | 50 | TMTV; OS Time-to-event; no fixed prediction horizon | HR | 1.275 (1.122–1.448) | 1.438 (1.208–1.713) |
| P045 | Weber et al., 2021 [33] C026 / CF024 | 50 | Lymph-node MTV; OS Time-to-event; no fixed prediction horizon | HR | 1.19 (1.022–1.384) | 1.266 (1.081–1.482) |
| P046 | Weber et al., 2021 [33] C026 / CF024 | 50 | Liver MTV; OS Time-to-event; no fixed prediction horizon | HR | 1.149 (1.001–1.318) | 1.178 (1.025–1.355) |
| P047 | Weber et al., 2021 [33] C026 / CF024 | 50 | TMTV; OS OS Cox-model concordance; no fixed horizon | Gonen-Heller concordance | 0.65 (CI NR) | 0.69 (CI NR) |
| P048 | Capobianco et al., 2021 [1] C027 / CF019 | 280 | TMTV; PFS 4-year discrimination | AUC | 0.63 (CI NR) | 0.69 (CI NR) |
| P050 | Capobianco et al., 2021 [1] C027 / CF019 | 280 | TMTV; OS 4-year discrimination | AUC | 0.65 (CI NR) | 0.68 (CI NR) |

n denotes the analysis population per method. NR, not reported; HR, hazard ratio; AUC, area under the receiver operating characteristic curve; CI, confidence interval; OS, overall survival; PFS, progression-free survival; FFR, freedom from recurrence; FFDM, freedom from distant metastasis; MTV, metabolic tumour volume; TMTV, total metabolic tumour volume; TTB, total tumour burden; TLG, total lesion glycolysis. *Nominal development cohort size; effective n may differ by horizon because unknown survival status was excluded. HRs describe follow-up associations and are not fixed-horizon risk ratios.

**Table S2 Comparison conditions and available uncertainty**

| ID | Transformation and threshold | Adjustment or model | Paired test and uncertainty | Source location and effect IDs |
|---|---|---|---|---|
| P001 | >210.2 vs ≤210.2 mL. AI: Manual-derived cutoff applied unchanged to TMTVcnn; expert: Minimum p-value in univariable Cox regression; optimized on TMTVman. | Univariable; no covariates. | No direct paired effect-difference test reported. Paired difference SE/CI and covariance not available in the extracted records. | Results p.5080; Fig. 7 p.5082<br>E002 / E001 |
| P020 | >290 vs ≤290 source-reported TLG units. AI: fixed from exploratory cohort; expert: fixed from exploratory cohort. | Univariable; no covariates. | No direct paired effect-difference test reported. Paired difference SE/CI and covariance not available in the extracted records. | Validation Results, p.4; Supplementary Fig. 1<br>E101 / E102 |
| P021 | >90 vs ≤90 mL. AI: manual-derived in-sample cutoff; expert: minimum log-rank p in development set. | Univariable; no covariates. | No direct paired effect-difference test reported. Paired difference SE/CI and covariance not available in the extracted records. | Table 4<br>E159 / E158 |
| P022 | >100 vs ≤100 mL. AI: manual-derived in-sample cutoff; expert: minimum log-rank p in development set. | Univariable; no covariates. | No direct paired effect-difference test reported. Paired difference SE/CI and covariance not available in the extracted records. | Table 4<br>E161 / E160 |

| ID | Transformation and threshold | Adjustment or model | Paired test and uncertainty | Source location and effect IDs |
| --- | --- | --- | --- | --- |
| P023 | >400 vs ≤400 SUVbw·mL. AI: manual-derived in-sample cutoff; expert: minimum log-rank p in development set. | Univariable; no covariates. | No direct paired effect-difference test reported. Paired difference SE/CI and covariance not available in the extracted records. | Table 4 E163 / E162 |
| P024 | >700 vs ≤700 SUVbw·mL. AI: manual-derived in-sample cutoff; expert: minimum log-rank p in development set. | Univariable; no covariates. | No direct paired effect-difference test reported. Paired difference SE/CI and covariance not available in the extracted records. | Table 4 E165 / E164 |
| P025 | Model AUC; TMTV with LDH category and brain metastases. Feature selection used outcome-optimized univariable results; nesting within each fold not established. | Multivariable model; TMTV, LDH category, brain metastases. | DeLong test, $p > 0.05$; exact p NR. Paired difference SE/CI and covariance not available in the extracted records. | Statistical analysis; Section 3.4; Table 5 E167 / E166 |
| P026 | Model AUC; TMTV with LDH category and brain metastases. Feature selection used outcome-optimized univariable results; nesting within each fold not established. | Multivariable model; TMTV, LDH category, brain metastases. | DeLong test, $p > 0.05$; exact p NR. Paired difference SE/CI and covariance not available in the extracted records. | Statistical analysis; Section 3.4; Table 5 E169 / E168 |

| ID | Transformation and threshold | Adjustment or model | Paired test and uncertainty | Source location and effect IDs |
|---|---|---|---|---|
| P027 | Model AUC; TMTV with LDH category and brain metastases. Feature selection used outcome-optimized univariable results; nesting within each fold not established. | Multivariable model; TMTV, LDH category, brain metastases. | DeLong test, p > 0.05; exact p NR. Paired difference SE/CI and covariance not available in the extracted records. | Statistical analysis; Section 3.4; Table 5<br>E171 / E170 |
| P028 | Model AUC; TMTV with LDH category and brain metastases. Feature selection used outcome-optimized univariable results; nesting within each fold not established. | Multivariable model; TMTV, LDH category, brain metastases. | DeLong test, p > 0.05; exact p NR. Paired difference SE/CI and covariance not available in the extracted records. | Statistical analysis; Section 3.4; Table 5<br>E173 / E172 |
| P029 | above vs at/below manual main-data median TTB. AI: manual main-data median; expert: manual main-data median. | Univariable; no covariates. | No direct paired effect-difference test reported. Paired difference SE/CI and covariance not available in the extracted records. | Results, publisher lines 209–212<br>E177 / E176 |
| P030 | above vs at/below fixed manual main-data median TTB. AI: fixed from main dataset; expert: fixed from main dataset. | Univariable; no covariates. | No direct paired effect-difference test reported. Paired difference SE/CI and covariance not available in the extracted records. | Results, publisher lines 209–212<br>E179 / E178 |

| ID | Transformation and threshold | Adjustment or model | Paired test and uncertainty | Source location and effect IDs |
|---|---|---|---|---|
| P039 | per 1 log10-unit increase. No dichotomizing cutoff for this estimate. | Univariable; no covariates. | No direct paired effect-difference test reported. Paired difference SE/CI and covariance not available in the extracted records. | Table 2; Results E227 / E226 |
| P040 | per 1 log10-unit increase. No dichotomizing cutoff for this estimate. | Univariable; no covariates. | No direct paired effect-difference test reported. Paired difference SE/CI and covariance not available in the extracted records. | Table 2; Results E229 / E228 |
| P041 | per 1 log10-unit increase. No dichotomizing cutoff for this estimate. | Univariable; no covariates. | No direct paired effect-difference test reported. Paired difference SE/CI and covariance not available in the extracted records. | Table 2 E231 / E230 |
| P042 | per 1 log10-unit increase. No dichotomizing cutoff for this estimate. | Univariable; no covariates. | No direct paired effect-difference test reported. Paired difference SE/CI and covariance not available in the extracted records. | Table 2 E233 / E232 |

| ID | Transformation and threshold | Adjustment or model | Paired test and uncertainty | Source location and effect IDs |
|---|---|---|---|---|
| P043 | per 1-unit increase in log10(TLG+1). No dichotomizing cutoff for this estimate. | Univariable; no covariates. | No direct paired effect-difference test reported. Paired difference SE/CI and covariance not available in the extracted records. | Abstract; Results E240 / E241 |
| P044 | per 1-unit increase in log2(MTV+1). No dichotomizing cutoff for this estimate. | Univariable; no covariates. | No direct paired effect-difference test reported. Paired difference SE/CI and covariance not available in the extracted records. | Results, Metabolic tumor volume E251 / E252 |
| P045 | per 1-unit increase in log2(MTV+1); no dichotomizing cutoff. | Separate forward-stepwise models. Other candidate organs: bone, liver, lung and soft-tissue. Method-specific MTV values. | No direct paired effect-difference test reported. Paired difference SE/CI and covariance not available in the extracted records. | Results, Organ-wise metabolic tumor volume E253 / E254 |
| P046 | per 1-unit increase in log2(MTV+1); no dichotomizing cutoff. | Separate forward-stepwise models. Other candidate organs: bone, lymph-node, lung and soft-tissue. Method-specific MTV values. | No direct paired effect-difference test reported. Paired difference SE/CI and covariance not available in the extracted records. | Results, Organ-wise metabolic tumor volume E255 / E256 |
| P047 | Cox-model concordance. No dichotomizing cutoff for this estimate. | Burden-based discrimination; no additional covariates recorded. | Bootstrap Gauss test, p = 0.19. Paired difference SE/CI and covariance not available in the extracted records. | Results, Metabolic tumor volume E257 / E258 |

| ID | Transformation and threshold | Adjustment or model | Paired test and uncertainty | Source location and effect IDs |
|---|---|---|---|---|
| P048 | 4-year discrimination. No dichotomizing cutoff for this estimate. | Burden-based discrimination; no additional covariates recorded. | No direct paired effect-difference test reported. Paired difference SE/CI and covariance not available in the extracted records. | Table 3 E260 / E262 |
| P050 | 4-year discrimination. No dichotomizing cutoff for this estimate. | Burden-based discrimination; no additional covariates recorded. | No direct paired effect-difference test reported. Paired difference SE/CI and covariance not available in the extracted records. | Table 3 E264 / E266 |

For P020, the cutoff is retained as 290 source-reported TLG units because the source table labels mL although conventional TLG units differ. For P044, the Results confidence interval for automated MTV is retained; the abstract repeats the manual interval. P045 and P046 record candidate organ variables, not a verified common final adjustment set. DeLong and bootstrap p values test differences and do not establish equivalence. Source locations refer to the report identified in Table S1; effect IDs link to Supplementary Data.

Table S2a Cohort dependence and prognostic validation in the comparative evidence

| Report / scope | Recorded evaluation and dependence | Implication for comparative inference |
|---|---|---|
| Nikulin 2026 [10] / PETAL | No overlap with another included outcome cohort identified. Threshold-based PFS HR lacks a confidence interval. | Cohort independence alone does not provide the uncertainty needed for comparative pooling. |
| Zschaeck 2025 [4] | Validation n=65 is a subset of the Nikulin 2023 external source population. | Do not count the linked comparisons as independent replication. |
| Dirks 2023 [37] | Eight records reuse the development cohort. Model AUCs use leave- | Internal prognostic evaluation; repeated horizons are not independent |

| Table S2a Cohort dependence and prognostic validation in the comparative evidence | | |
|---|---|---|
| **Report / scope** | **Recorded evaluation and dependence** | **Implication for comparative inference** |
| | one-out cross-validation. The separate n=31 test cohort has no paired manual comparison. | validation cohorts. Nominal n=69 is not verified at every horizon. |
| Nikulin 2023 [3] | Main and external outcome subsets differ, but external source patients overlap Zschaeck. Shared TCIA data also link Leung; exact analytic overlap is unenumerated. | An external source dataset does not establish independence across reports or validation of an unchanged prognostic procedure. |
| Han 2022 [32] | Four records reuse one thymic cohort; recurrence and OS are distinct. Only eight deaths were recorded. | Separate outcomes are not independent replication; OS precision is limited. Fixed-procedure external validation is not established by these records. |
| Borrelli 2022 [2] | One diagnostically mixed cohort with patient holdout; test centres contributed training data. | Patient holdout is not centre-independent validation of the prognostic procedure. |
| Weber 2021 [33] | Four records reuse one breast cohort. Organ-specific AI and manual models use separate forward-stepwise selection. | A common fixed adjustment set is not established; repeated analyses are not independent replication. |
| Capobianco 2021 [1] / REMARC | REMARC overlaps Girum REMARC analyses outside the aligned set. Girum LNH073B is a separate cohort. | Catalogue-family identifiers do not establish patient independence or a replicated common estimand. |
| Kovacs 2026 [27] / additional comparison outside aligned set | Manual- and AI-volume recurrence models are fitted separately and evaluated in the same dataset. Contour targets differ. | Not fixed-coefficient external validation. Paired-difference uncertainty for the non-inferiority inference is unverified. |

This focused table summarizes existing records, not a new validation assessment of all 35 reports. Tables S1–S2 and Supplementary Data retain source locations and comparison identifiers; Kovacs [27] qualifications are detailed in S10–S11. Unreported or unresolved independence and threshold-transfer conditions are not treated as confirmed external prognostic validation.

Restricted pooling assessment

Lymphoma provides more than one source cohort but not a compatible replicated estimand: PETAL contributes a threshold-based PFS HR without its confidence interval, whereas REMARC contributes four-year AUCs. Other REMARC HRs use method-specific cutoffs, and projection or dissemination surrogates do not measure the same contrast. REMARC reuse also prevents treating linked reports as independent.

Head and neck comparisons share source patients, use different burden measures and outcomes, and lack sufficient paired uncertainty. The independent Kovacs [27] cohort compares different contour targets and recurrence models, so it is outside this aligned stratum. Each remaining aligned disease group contains one report; multiple outcomes, biomarkers or prediction horizons from that report do not provide independent replication.

The extracted records therefore do not identify a disease-specific set of independent, compatible contrasts with sufficient comparative uncertainty to pool an AI-versus-expert effect. This finding applies to the available records and does not exclude every conceivable meta-analysis. Separate AI and expert estimates were not treated as independent. No individual-patient data or additional investigator data were obtained.

Sources for Tables S1 and S2 are listed in the main manuscript reference list [1–4,10,32,33,37].

**S10 Report-level appraisal coverage and factor-analysis scopes**

Table S3 maps all 35 included reports to the recorded appraisal routes. Counts denote assessment units or records, not independent studies, models or patient groups. The confirmed totals remain 62 QUIPS units in 33 reports, six Form S scopes in five reports and 119 model-assessment records in ten reports. Form S is a review-specific, unvalidated segmentation instrument; model records use the framework based on PROBAST+AI described in S6. A zero indicates no recorded assessment under that route, not absence of prognostic evidence. Gutsche et al. [19] contribute two finalized outcome-specific scopes and Küper et al. [20] contribute four finalized factor/outcome scopes, adopted on 15 October 2026. The six Q01–Q06 scopes in Table S4 were also adopted on 15 October 2026.

**Table S3. Report-level appraisal coverage**

| Report ID | Report | Tier | QUIPS units | Form S scopes | Model records | Factor-scope status |
|---|---|---|---|---|---|---|
| R001 | Sachpekidis et al., 2026 [35] | Contextual | 3 | 0 | 0 | Recorded coverage |
| R002 | Jamet et al., 2026 [41] | Contextual | 2 | 0 | 8 | Recorded coverage |
| R003 | Nikulin et al., 2026 [10] | Core | 1 | 1 | 0 | Recorded coverage |
| R004 | Xue et al., 2026 [8] | Contextual | 1 | 0 | 4 | Recorded coverage |
| R005 | Kovacs et al., 2026 [27] | Core | 2 | 1 | 16 | Two factor/outcome and four development scopes adopted 15 October 2026 |
| R006 | Wang et al., 2025 [21] | Contextual | 4 | 2 | 7 | Recorded coverage |
| R007 | Ye et al., 2026 [7] | Contextual | 1 | 0 | 0 | Recorded coverage |
| R008 | Zang et al., 2026 [5] | Contextual | 1 | 0 | 3 | Recorded coverage |
| R009 | Beintner-Skawran et al., 2025 [38] | Contextual | 1 | 0 | 0 | Recorded coverage |
| R010 | Sachpekidis et al., 2025 [36] | Contextual | 2 | 0 | 0 | Recorded coverage |
| R011 | Kovacs et al., 2025 [28] | Contextual | 1 | 0 | 0 | Recorded coverage |
| R012 | Lv et al., 2025 [29] | Contextual | 2 | 0 | 0 | Recorded coverage |
| R013 | Zschaeck et al., 2025 [4] | Core | 2 | 1 | 0 | Recorded coverage |
| R014 | Galli et al., 2025 [22] | Contextual | 1 | 0 | 0 | Recorded coverage |
| R015 | Sachpekidis et al., 2024 [42] | Contextual | 2 | 0 | 0 | Recorded coverage |
| R016 | Dirks et al., 2023 [37] | Core | 2 | 1 | 26 | Recorded coverage |

| Report ID | Report | Tier | QUIPS units | Form S scopes | Model records | Factor-scope status |
|---|---|---|---|---|---|---|
| R017 | Kendrick et al., 2023 [39] | Core | 1 | 0 | 0 | Recorded coverage |
| R018 | Nikulin et al., 2023 [3] | Core | 2 | 0 | 0 | Recorded coverage |
| R019 | Kendrick et al., 2022 [40] | Contextual | 1 | 0 | 0 | Recorded coverage |
| R020 | Jemaa et al., 2022 [9] | Core | 4 | 0 | 15 | Training846:2; full1268:2; overlapping cohort |
| R021 | Girum et al., 2022 [23] | Core | 4 | 0 | 0 | Recorded coverage |
| R022 | Han et al., 2022 [32] | Core | 2 | 0 | 0 | Recorded coverage |
| R023 | Jiang et al., 2022 [24] | Contextual | 2 | 0 | 0 | Recorded coverage |
| R024 | Borrelli et al., 2022 [2] | Core | 1 | 0 | 0 | Recorded coverage |
| R025 | Polymeri et al., 2021 [6] | Contextual | 1 | 0 | 0 | Recorded coverage |
| R026 | Weber et al., 2021 [33] | Core | 1 | 0 | 0 | Recorded coverage |
| R027 | Capobianco et al., 2021 [1] | Core | 2 | 0 | 0 | Recorded coverage |
| R028 | Polymeri et al., 2020 [13] | Contextual | 1 | 0 | 0 | Recorded coverage |
| R029 | Leung et al., 2024 [30] | Contextual | 2 | 0 | 8 | Two factor/outcome scopes finalized 15 October 2026 |
| R030 | Luo et al., 2026 [25] | Contextual | 0 | 0 | 8 | Model route; abstract only |
| R031 | Nafeli et al., 2026 [31] | Contextual | 0 | 0 | 24 | Model route in current extraction |
| R032 | Liang et al., 2026 [34] | Contextual | 2 | 0 | 0 | Recorded coverage |
| R033 | Gutsche et al., 2023 [19] | Core | 2 | 0 | 0 | 2 QUIPS scopes finalized 15 October 2026 |
| R034 | Küper et al., 2024 [20] | Contextual | 4 | 0 | 0 | 4 QUIPS scopes finalized 15 October 2026 |

| Report ID | Report | Tier | QUIPS units | Form S scopes | Model records | Factor-scope status |
|---|---|---|---|---|---|---|
| R035 | Le Goff et al., 2023 [26] | Contextual | 2 | 0 | 0 | 2 QUIPS scopes finalized 15 October 2026 |
| Total | 35 reports | 13 Core; 22 Contextual | 62 | 6 | 119 | Routes overlap |

**Additional factor-appraisal scopes**

Kovacs (2026) [27], Jemaa [9] and Leung [30] report tumour-burden associations in addition to model-performance results. Table S4 lists six factor/outcome scopes (Q01–Q06), adopted by the researcher on 15 October 2026 with 36 domain judgments. Leung MTV and TLA remain separate factor scopes sharing the same 74-patient cohort. These six scopes add three reports to QUIPS coverage; they are not six independent cohorts. Jemaa's separate n=1268 comparative analyses are not covered by its n=846 training-sample assessments.

**Table S4. Additional finalized QUIPS scopes and linked factor estimates**

| Scope / report | Factor and outcome | n | Effect ID | HR (95% CI) and p | Analysis context |
|---|---|---|---|---|---|
| Q01 / R020 | aTMTV>Q3; PFS | 846 | E184 | 1.31 (0.98–1.74); p=.06 | Training adjusted coefficient |
| Q02 / R020 | aTMTV>Q3; OS | 846 | E185 | 1.18 (0.83–1.67); p=.35 | Training adjusted coefficient |
| Q03 / R029 | MTV continuous; OS | 74 | E007/E009 | 0.99 (0.97–1.02); p=.57 | Adjusted; univariable 1.01 (1.01–1.02), p<.001 |
| Q04 / R029 | TLA continuous; OS | 74 | E008/E010 | 1.00 (1.00–1.00), rounded; p=.27 | Adjusted; rounded HR/CI not exact zero effect |
| Q05 / R005 | AI-PET-GTV log($cm^3$); LRF | 540 | E034 | 1.43 (1.19–1.71); p<.001 | Cause-specific adjusted coefficient |
| Q06 / R005 | AI-PET-GTV log($cm^3$); DM | 540 | E036 | 2.62 (1.9–3.62); p<.001 | Cause-specific adjusted coefficient |

Jemaa [9] (R020; Q01–Q02). The n=846 training-cohort aTMTV coefficients for PFS and OS are adjusted estimates from Table 2 after variable screening, LASSO selection and Cox refitting; neither adjusted association meets the 0.05 threshold. These coefficients are not holdout validation results. QUIPS review must address selection into the imaging-evaluable population, follow-up completeness, factor measurement and cutoff derivation, endpoint ascertainment, confounding and selection-related analysis bias. The 48.2-month median follow-

up is reported for the overall cohort and is not a training-specific value. GOYA defines PFS from randomization to progression, relapse or all-cause death, and OS to all-cause death (NCT01287741). Analysis-specific follow-up losses and missing-data handling remain unresolved.

Supplementary Table 4 of Jemaa [9] reports four same-patient manual-versus-automated TMTV comparisons in 1268 patients (PFS/OS, median/Q3). On 15 October 2026, the researcher adopted Core classification and two additional outcome-specific QUIPS scopes, with median/Q3 retained as linked contrasts within each scope. The comparisons remain outside the 23 aligned records because matching quantile labels do not establish identical numerical cutoffs; adjustment is unspecified and Table 2 covariates are not transferred. Similar HRs do not establish equivalence or independent external validation.

Leung [30] (R029; Q03–Q04). MTV and TLA concern OS in the same n=74 head-and-neck cancer cohort. The univariable MTV HR is 1.01 (95% CI 1.01–1.02; p<.001), whereas the adjusted HR is 0.99 (0.97–1.02; p=.57). Rounded TLA values of 1.00 are not exact evidence of no effect. Retain both univariable and adjusted estimates, and decide whether a shared cohort/outcome assessment with predictor-specific subrecords is sufficient. Event counts, follow-up, missingness, clinical adjustment, and overlap or collinearity among imaging predictors and composite scores need explicit consideration.

Kovacs [27] (R005; Q05–Q06). The AI-PET-GTV coefficients use log-transformed volume and separate cause-specific models for loco-regional failure (LRF) and distant metastasis (DM), with death without disease as a competing event. Manual-volume estimates E033 and E035 are linked comparators within the same cohort/outcome contexts. The supplied code separately fits manual and AI models and evaluates them in the same n=540 cohort; this is apparent evaluation after refitting, not fixed-coefficient external validation. The LRF formula includes smoking, subsite, T and N stage, log volume and cisplatin stratification; the DM formula substitutes HPV group for subsite. Age and performance status enter the competing-death formula rather than these LRF/DM formulas. QUIPS review must retain these endpoint-specific distinctions and separate censoring from attrition.

These source checks informed the appraisal scopes subsequently adopted on 15 October 2026 (S15–S17). Analysis-specific follow-up and missingness limitations remain explicit. Luo [25] retains its abstract-only model assessment; the adopted Nafeli [31] revisions are recorded in S11. Original judgments are preserved in the audit trail, and current finalized counts are reported in S10 and S17.

Source locations: Jemaa et al. (2022) [9], main Table 2 and Supplementary Table 4 (https://pmc.ncbi.nlm.nih.gov/articles/PMC9373298/); Leung et al. (2024) [30], main Table 2 and supplement (J Nucl Med. 65:643; https://jnm.snmjournals.org/content/65/4/643); Kovacs et al. (2026), main Table 1 and supplementary cause-specific model code (doi:10.1186/s13550-

026-01377-0 [27]). Finalized judgments and linked estimates: Supplementary Assessment Details and Supplementary Data. GOYA registry: https://clinicaltrials.gov/study/NCT01287741.

**S11 Comparison with the official PROBAST+AI form and guidance**

The complete 2025 PROBAST+AI [15] form and E&E Light were compared with the existing framework. All 34 phase-specific concepts match, and the 119 overall labels agree with aggregation of their recorded domains. This establishes structural consistency, not answer validity. The 2064 stored responses are not necessarily applicable questions.

On 15 October 2026, the researcher adopted 198 item-response changes and 45 retain-response recommendations, resolving all 243 audit flags. The changes comprise 84 NA, 84 NI, 18 N and 12 PN responses. Reasoned analysis-domain revisions changed 22 evaluation records from Unclear to High (four Jemaa [9] and 18 Nafeli [31] records). The inventory remains 39 development and 80 evaluation records; development-quality judgments are 17 High and 22 Unclear, and evaluation-risk judgments are 67 High and 13 Unclear. These counts describe assessment records, not independent models or studies. Original responses and rationales are preserved in the audit trail. NA routing does not demonstrate absence of overfitting or leakage, and NI was not automatically converted to High.

Kovacs [27] Fig. S1 shows apparent calibration only for three-year LRF in both models. Code requests other calibration outputs but does not establish their reported results. Two refitted competing-risk systems yield four target model/outcome development scopes, sharing 16 evidence records; these were adopted as four development-quality assessments with Unclear judgments. They do not change the existing 12 High-risk evaluation records. The non-inferiority code uses the manual-model CI-derived SE rather than a paired-difference SE. Reported values are preserved, with this uncertainty limitation recorded.

The original 115 imbalance items were mapped to 15 shared-evidence groups. Stratified cross-validation, score weights, weighted log-rank tests and stage matching were not automatically interpreted as outcome-class balancing. Missing reporting did not establish non-use or NA. The researcher also adopted the review-level PICOTS clarification, applicability explanations and four Kovacs [27] development assessments on 15 October 2026. The resulting inventory comprises 119 records (39 development and 80 evaluation), 2064 item responses, 476 quality or risk-of-bias domain judgments and 357 applicability explanations. Development quality was High in 17 and Unclear in 22 records; evaluation risk of bias remained High in 67 and Unclear in 13. Overall applicability concern was High in eight and Unclear in 111 records. These are assessment records, not independent models or patient cohorts. This targeted reconciliation does not establish independent duplicate assessment or complete source-level verification of every item. Form S remains a review-specific,

unvalidated instrument. Source: Moons et al. [15], BMJ 2025;388:e082505, Supplementary Tables 3–4.

**S12 Additional full-text checks during revision**

Additional source checking of ten reports retained in the separate revision-stage follow-up set supported nine E1 exclusions, adopted by the researcher on 15 October 2026, because the reported PET measurement procedures had no learning-based component, and one E2 exclusion because the AI-derived burden itself was not evaluated prognostically. The researcher also adopted the E2 exclusion. These ten decisions belong to the revision-stage follow-up branch and are included in its 291 exclusions. Küper et al. (doi:10.7150/thno.96738 [20]) were adopted as Contextual evidence by the researcher on 14 October 2026. Its four QUIPS scopes were finalized on 15 October 2026.

For the included Küper [20] report (R034; C034; catalogue family CF030), whole-body PSMAvol analyses were extracted separately from organ-specific and composite-score analyses. MICIIS is explicitly linked to a prior method using neural-network anatomical masks. Eligibility is supported by this cited-method linkage, not an independent audit of its implementation. No paired expert-versus-AI prognostic comparison was identified. Inclusion was adopted by the researcher; extraction values remain source-checked assistant records, and the four QUIPS scopes were finalized by the researcher on 15 October 2026.

The ten reports were checked against uploaded full texts, including the supplementary Methods for Wang et al. (10.1002/hon.70118 [49]). Adopted E1 exclusions apply to 10.1002/hon.70118 [49]; 10.1097/RLU.0000000000005863 [50]; 10.1182/blood.2024024965 [51]; 10.1016/j.ejrad.2023.111198 [52]; 10.1007/s00277-023-05379-z [53]; 10.1097/MNM.0000000000001713 [54]; 10.1007/s11604-022-01369-9 [55]; 10.1097/MNM.0000000000001669 [56]; and 10.1007/s00277-022-04988-4 [57]. The adopted E2 exclusion applies to 10.1007/s10278-026-02224-3 [58]: its survival head uses shared image features, while the report does not establish a burden-specific prognostic analysis. Dice comparisons by vital status were not interpreted as tumour-burden effects.

Küper et al. [20] analyzed 73 patients at Essen who had paired PSMA PET examinations without LuPSMA treatment; 26 deaths were reported. Comparison with the institutions and populations in the existing prostate-cancer extraction records identified no evidence of cohort overlap, although individual-level overlap was not audited. Whole-cohort baseline body-weight-adjusted PSMAvol was associated with OS (HR 3.920, 95% CI 2.127–7.224). The change estimate was 10.481 (2.051–53.560) univariably and 6.502 (1.018–41.552) after adjustment for PPP and RECIP. These continuous, body-weight-adjusted estimates must not be interpreted as per-mL effects. The survival time origin and the direction of the absolute difference were not sufficiently specified to infer additional definitions.

Table 5 labels the bone-volume change HR as 31.106 (5.623–172.100) univariably and 37.270 (5.460–254.400) in a model containing other regional changes and age. The abstract uses inconsistent baseline/response labels and a response HR of 32.27. These discrepancies were retained rather than corrected by inference. Figure 3 describes OS but labels its time axis as time to progression; no numerical OS results were digitized from that figure.

Four finalized QUIPS scopes address baseline PSMAvol, PSMAvol change, PSMA organ score and RECIP PD versus non-PD associations with OS. Their 24 domain judgments were adopted by the researcher on 15 October 2026. The principal concerns are clinically selected repeat imaging, incomplete reporting of follow-up and time origin, treatment and disease-stage confounding, sparse events and exploratory analyses. Composite-score hazard ratios and Kaplan–Meier comparisons were treated as prognostic associations, not demonstrations of individual-risk predictive performance; no new PROBAST-AI model-performance forms were added for these association-only results.

The separate extraction preserves nine whole-body, twelve composite-score and ten regional estimates, including nonsignificant results. They are correlated analyses, not independent cohorts. The adopted report is included in the 35-report catalogue. The earlier 100-assessed branch is historical; after Le Goff [26] reassessment the original branch contains 101 assessed and 69 excluded reports. The revision-stage inclusion is shown separately in S24. Finalized appraisal denominators remain unchanged. Sources: Küper et al. [20], Theranostics 2024;14:3623–3633, Tables 3–5 and Figure 3; Seifert et al., J Nucl Med 2020;61:1786–1792, doi:10.2967/jnumed.120.242057 [59].

**S13 Reclassification of PM00774 during revision**

On 14 October 2026, the researcher adopted inclusion of Gutsche et al. (PM00774; doi:10.2967/jnumed.123.265725 [19]) as Core evidence. The previous E2 rationale stated that AI-derived MTV survival analyses were absent. Main-text pages 1598–1600, Figure 5 and supplement page 5 document such analyses. The historical decision is preserved and superseded; the linked conference abstract EM26-L644189101 is not counted as another included report.

In a small retrospective glioma response cohort, automated and physician-assessed $^{18}$F-FET PET MTV changes both showed OS separation, whereas PFS separation was not statistically significant (network, P = 0.39; physician, P = 0.12). The report described 33 patients, but the initial risk sets in Figure 5 totaled 32. These unadjusted findings do not establish independent prognostic value or interchangeability. Independence of the response cohort from segmentation development and the handling of survival time relative to post-treatment measurement were not established.

The report is registered as R033/C033/CF029, a separate catalogue family based on the current report and cohort mapping, not a claim of independent prognostic validation. The parent Ceccon study supplied the response cohort; its adjusted effect estimates are not assigned to the AI reanalysis. The exact histology of the 33-patient subset and the reason for the 33/32 discrepancy remain unresolved. Parent survival definitions start at diagnosis, whereas change is measured after treatment; the AI reanalysis does not independently establish landmark or time-dependent handling.

Two qualitative outcome comparisons and four log-rank result records were added. The 23 aligned HR/AUC/concordance comparisons remain unchanged. After the separate inclusion decision, the researcher adopted both QUIPS scopes and all twelve domain judgments on 15 October 2026. This was verification of AI-assisted proposals, not independent duplicate review.

**S14 Adoption of Küper as Contextual evidence**

The researcher adopted Contextual inclusion on 14 October 2026. The report is registered as R034/C034/CF030. Catalogue comparison identified no matching cohort among the existing 33 reports; this is not patient-level overlap verification or independent prognostic validation. Thirty-one association estimates are preserved: nine whole-body, twelve composite-score and ten regional results. One cohort record, one measurement-method record, nine burden/composite records and one OS outcome record were added. HSPC and CRPC are nested subgroups of the same cohort, not separate cohort families. The 23 aligned comparisons and two qualitative Gutsche [19] comparisons are unchanged. Subsequent researcher adoption of the two Gutsche and four Küper [20] QUIPS scopes increased the finalized inventory to 52 units; adoption of Q01–Q06 and two full-cohort Jemaa [9] scopes subsequently brought the total to 60.

The revision search export dated 13 October 2026 contained 2402 retained records. After linking 18 duplicates, 2384 active records remained: 2092 screening exclusions and 292 follow-up records (291 exclusions and one Contextual inclusion, Küper et al. [20]). All record-level dispositions were adopted by the author on or before 15 October 2026. The five duplicates within the 297 raw follow-up rows are included in the 18 links and are not subtracted twice. Thus, the revision branch comprises 2383 exclusions and one inclusion. These are AI-assisted, author-adopted decisions, not independent duplicate screening; source access ranged from abstracts to complete reports. Revision exclusions are not added to the original 69 full-text exclusions.

## S15 Researcher-adopted QUIPS judgments for Gutsche and Küper

On 15 October 2026, the researcher adopted the two Gutsche [19] MTV-change scopes (OS and PFS) and four Küper [20] OS association scopes (baseline PSMAvol, PSMAvol change, PSMA organ score and RECIP). For each Gutsche scope, the six domain ratings were Moderate, Moderate, Moderate, Moderate, High and High. For each Küper scope, the ratings were High, Moderate, Moderate, Moderate, High and High, in the order participation, attrition, factor measurement, outcome measurement, confounding, and analysis/reporting. After adoption of the six Q01–Q06 scopes and the two Jemaa [9] full-cohort scopes, the interim inventory contained 60 units from 32 reports, with 37 high-confounding and 48 high-analysis/reporting judgments. Subsequent adoption of the two Le Goff [26] scopes brought the current inventory to 62 units from 33 reports, with 37 high-confounding and 50 high-analysis/reporting judgments.

The full 36-domain addendum retains source locations, rationale, original proposals and dated researcher adoption. For Gutsche [19], high analysis/reporting risk concerns time-related analysis uncertainty, small samples, multiple analyses and reporting inconsistencies; nonsignificant PFS is not itself a bias criterion. Missing HR/CI and lack of an equivalence test constrain interpretation. For Küper [20], scope-specific concerns distinguish baseline confounding, time origin for response/RECIP and selection of organ-score plot cutoffs. Published-source discrepancies are retained; adopting the ratings does not resolve them.

## S16 Adoption of the six additional factor analysis scopes

On 15 October 2026, the researcher retained and adopted all six scopes Q01–Q06 and 36 domain judgments. Jemaa [9] aTMTV–PFS: High, Moderate, Moderate, Moderate, High, High; Jemaa aTMTV–OS: High, Moderate, Moderate, Low, High, High. Leung [30] MTV–OS and TLA–OS: Moderate, Moderate, Moderate, Moderate, High, High. Kovacs [27] AI-PET-GTV–LRF and –DM: Moderate, Moderate, Low, Moderate, Moderate, Moderate. Domain order is participation, attrition, factor measurement, outcome measurement, confounding, and analysis/reporting. The interim inventory was 60 units in 32 reports (360 domain judgments), with 37 high-confounding and 48 high-analysis/reporting judgments. Following addition of the two Le Goff [26] scopes, the current inventory is 62 units in 33 reports (372 domain judgments), with 37 high-confounding and 50 high-analysis/reporting judgments.

These are factor/outcome assessment units, not independent cohorts. The two Leung [30] factors share evidence but remain separately recorded, consistent with the Küper [20] factor-specific scopes. Kovacs [27] performance-validation and noninferiority concerns are not automatically assigned to its factor-HR analyses. Jemaa [9]'s n=1268 median/Q3

comparative results remain distinct from the n=846 selected training coefficients; their two outcome-specific scopes and Core reclassification were separately adopted on 15 October 2026.

### S17 Jemaa full cohort comparisons and adopted appraisal

Supplementary Table 4 of Jemaa [9] reports four same-patient manual-versus-automated TMTV comparisons in 1268 patients (PFS/OS, median/Q3). On 15 October 2026, the researcher adopted Core classification and two additional outcome-specific QUIPS scopes, with median/Q3 retained as linked contrasts within each scope. The comparisons remain outside the 23 aligned records because matching quantile labels do not establish identical numerical cutoffs; adjustment is unspecified and Table 2 covariates are not transferred. Similar HRs do not establish equivalence or independent external validation.

The two full-cohort QUIPS scopes were adopted with ratings High/Moderate/Moderate/Moderate/High/High for PFS and High/Moderate/Moderate/Low/High/High for OS, in the order participation, attrition, factor measurement, outcome measurement, confounding, analysis/reporting. Imaging selection was associated with survival, follow-up completeness remains uncertain, and Supplementary Table 4 does not specify adjustment. The four contrasts share one cohort and two outcomes; they are not four independent studies. Current totals are 35 reports (13 Core/22 Contextual), 62 QUIPS units from 33 reports, 37 high-confounding and 50 high-analysis/reporting units. All 23 aligned comparison records remain unchanged.

Supplementary Table 4 results, AI versus manual HR (95% CI): PFS median 1.70 (1.39–2.07) versus 1.76 (1.42–2.17); PFS Q3 1.94 (1.58–2.38) versus 1.96 (1.60–2.41); OS median 1.72 (1.34–2.21) versus 1.83 (1.38–2.39); OS Q3 2.03 (1.58–2.62) versus 2.11 (1.64–2.72). Eight effect records and four comparison links were added; a manual TMTV companion record was registered without adding a cohort.

### S18 Core only sensitivity synthesis

On 15 October 2026, the researcher adopted the descriptive restriction analysis. The analysis was initially adopted when the catalogue contained 34 reports (13 Core and 21 Contextual). The subsequent Contextual inclusion of Le Goff [26] brought the current catalogue to 35 reports (13 Core and 22 Contextual) without changing the restricted sets: 13 Core reports and 23 aligned comparisons from eight reports. The Core set contains 57 historical comparison links plus two qualitative Gutsche [19] links and four additional Jemaa [9] links; these 63 records are not independent studies. The aligned set comprises 16 HR, six AUC and one Gonen–Heller concordance comparison.

Four Dirks [37] AUC comparisons (P025–P028) evaluate multivariable models and were considered separately. The remaining 19 records comprise 16 HR comparisons, two burden-based AUC comparisons and one concordance comparison; they are not all univariable analyses. Evidence of prognostic association persisted in several settings, without establishing general equivalence or superiority. Han [32] OS estimates and Gutsche [19] PFS findings were retained without selection for statistical significance.

The restrictions did not resolve differing scales, endpoints, horizons, repeated analyses, shared patients or incomplete uncertainty for paired differences. R013/R018 share a source programme, R021/R027 overlap in REMARC, and Jemaa [9] training and full-cohort analyses overlap. No patient totals or independent-study totals were obtained by summing comparison records. This was a descriptive evidence restriction, not a pooled-effect, risk-of-bias-exclusion or external-validation-only analysis.

The six previously E2-excluded reports were rechecked and retained as excluded. The recovered records PM00631 and PM01516 completed the six-report identity reconciliation. The melanoma report EM00607 was linked to its published version (10.1016/j.ctro.2025.101063 [60]); this version correction did not create an additional report. The detailed decision register preserves source locations and reasons.

**S19 Adopted PICOTS and applicability**

The revision-stage PICOTS clarification covers disease- and treatment-specific prognosis using AI-derived PET tumour burden or composite models and expert or clinical comparators. Outcomes include survival, progression, recurrence and treatment response. Baseline and updated predictions are distinct; concurrent PSA classification is contextual. This clarification is neither prospectively registered nor a new eligibility exclusion.

Applicability was assessed separately from development quality and evaluation risk of bias. Clinical or molecular predictors did not alone make a composite model inapplicable. Outcome applicability was distinguished from follow-up missingness and validation limitations. Uncertain predictor availability, differing GTV/MTV constructs, updated-prediction landmarks and transportability were retained in the domain explanations. High applicability concern remains for two concurrent-PSA classification records and six stage-mixed Nafeli [31] evaluations.

Four Kovacs [27] development scopes cross manual/AI volume with LRF/DM; 1/3/5-year horizons share scopes. Sixteen evidence items yield 64 responses, 16 quality-domain judgments and 12 applicability explanations. Development quality and applicability are Unclear in all four; existing evaluation risk remains High.

## S20 Operational criteria and boundary cases

The archived decisions D-16, D-18 and D-19 (29 August 2026) separate report classification, paired-comparison reporting and eligibility for quantitative synthesis. The current evidence map links all 35 reports to the adopted tiers, source-located extraction and comparison records: 13 Core and 22 Contextual. This mapping clarifies implementation during revision; it does not establish that all later boundary interpretations were prospectively specified. Operationally, Core required eligible AI-derived PET tumour-burden measurement, an expert comparator in the same cohort and results permitting direct prognostic comparison; D-18 also allowed classification agreement. Other eligible prognostic reports were Contextual, subject to the documented measurement-construct boundary described below. Tier membership does not depend on statistical significance or a low-risk-of-bias rating. The historical principles and revision-stage clarifications are identified separately to make the adopted classifications auditable.

Core membership does not require every comparison to be aligned. Kendrick contributes same-cohort response-classification evidence, while its nested unequal-sample survival estimates remain separate. Kovacs [27] compares different GTV targets; Girum [23] compares 2D surrogates with 3D measurements. The four additional Jemaa [9] comparisons have unresolved numerical cutoff alignment, and Gutsche [19] contributes qualitative OS/PFS comparisons. These records remain outside the 23 aligned comparisons from eight reports.

Polymeri 2020 [13] (R028) reports AI and manual prognostic results in the same 43 patients (Table 5; P052–P060). Its Contextual classification cannot therefore be explained by absent expert comparison. The CT-derived whole-prostate mask yields anatomical volume and uptake-based measures rather than lesion-defined tumour burden. We retain its adopted Contextual tier and make this construct boundary explicit. The archived D-169 decision establishes the tier, while this more specific rationale is a revision-stage clarification.

Absence of paired covariance does not itself remove a report from Core. Under D-18, results may be presented side by side without calculating an unsupported difference standard error. D-19 separately requires compatible effects, valid uncertainty, clinical similarity and manageable dependence before pooling. Neither Core membership nor similar point estimates establishes equivalence.

## S23 Le Goff reassessment and current catalogue

Le Goff et al. [26] (2023; PM00843) were adopted as Contextual evidence after full-text reassessment during revision. Both original reviewers had excluded the record at title/abstract screening; their reason cells were blank, so no historical E code can be assigned. Reversal adds one assessed and one included report to the original database branch, without

changing the 69 full-text exclusions. The linked conference record is not counted as another included report. Its conference-abstract record was excluded under E4 on researcher adoption.

CF031 is a provisional catalogue family for the Créteil 2017–2020 outcome cohort. Catalogue comparison identified no confirmed overlap with the included PETAL, GOYA, REMARC/LNH073B, Chinese, Italian CAR-T or Canadian follicular-lymphoma cohorts. This is not a patient-level independence audit; overlap with the cited segmentation-development data remains unresolved.

The researcher adopted two Le Goff [26] QUIPS scopes on 15 October 2026: TMTV–PFS and combined ctDNA/TMTV–PFS. Both were Moderate in the first five domains and High for statistical analysis/reporting. The scopes share patients. Current totals are 62 QUIPS units from 33 reports; Form S and PROBAST+AI inventories are unchanged.

Three overlapping TMTV PFS hazard-ratio records were added as E359–E361; the R-CHOP-like subgroup analysis size is unreported. The Table 2 univariable p value is a log-rank test. Combined ctDNA/TMTV group survival is retained as descriptive evidence, separately from burden-factor effects. The current catalogue has 35 reports (13 Core, 22 Contextual). The Core-only and 23 aligned-comparison syntheses are unchanged.

On 15 October 2026, the researcher adopted the companion conference record EM26-L635343157 as an E4 exclusion while retaining journal R035. The revision follow-up set remains 292 active records, with 292 closed and none open (291 exclusions and one inclusion). The original database full-text exclusion total remains 69. The two additional QUIPS scopes add 12 domain judgments, giving 372; high confounding remains 37 and high statistical-analysis/reporting risk increases to 50. No new PROBAST+AI or Form S record was added.

The revision search export dated 13 October 2026 contained 2402 retained records. After linking 18 duplicates, 2384 active records remained: 2092 screening exclusions and 292 follow-up records (291 exclusions and one Contextual inclusion, Küper et al. [20]). All record-level dispositions were adopted by the author on or before 15 October 2026. The five duplicates within the 297 raw follow-up rows are included in the 18 links and are not subtracted twice. Thus, the revision branch comprises 2383 exclusions and one inclusion. These are AI-assisted, author-adopted decisions, not independent duplicate screening; source access ranged from abstracts to complete reports. Revision exclusions are not added to the original 69 full-text exclusions.

**S24 Final revision disposition and source limitations**

The revision search export dated 13 October 2026 contained 2402 retained records. After linking 18 duplicates, 2384 active records remained: 2092 screening exclusions and 292 follow-up records (291 exclusions and one Contextual inclusion, Küper et al. [20]). All record-level dispositions were adopted by the author on or before 15 October 2026. The five duplicates within the 297 raw follow-up rows are included in the 18 links and are not subtracted twice. Thus, the revision branch comprises 2383 exclusions and one inclusion. These are AI-assisted, author-adopted decisions, not independent duplicate screening; source access ranged from abstracts to complete reports. Revision exclusions are not added to the original 69 full-text exclusions.

The original database searches were conducted on 29 August 2026, and the supplementary-search record closed on 9 September 2026. The revision export is dated 13 October 2026; record-level dispositions were finalized on or before 15 October. Revision reassessment retained the original temporal eligibility boundary of 9 September, based on first online publication. It therefore does not constitute a comprehensive search update through the revision date, and later eligible publications may have been missed. No additional databases or broader searches were undertaken during finalization. The retained audit documents 5438 Embase bibliographic records and 31 PubMed additions (1817 versus the original 1786 PMIDs), with 3050 previously retrieved records and 17 current-search duplicates removed before the 2402-record queue. Sixty trial-registry records were kept separately. Exact revision-query reproducibility is limited to the archived source records. Web of Science, Scopus, and IEEE Xplore were not searched because institutional subscription access was unavailable when the review was initiated. This restriction may have reduced coverage of technical and multidisciplinary literature, particularly conference proceedings. The PubMed and Embase searches and supplementary searching do not fully resolve this coverage limitation. The missing Google Scholar query/settings and row-level excluded-record tracker remain limitations, so the branch accounting is not a claim of exhaustive cross-route deduplication.

The final method review distinguishes learned measurement components from downstream prognosis models. Under the operational eligibility rule, a learned anatomical mask can contribute to PET-burden measurement, whereas FLAB without a separately trained learning component, gradient-based contouring, or fixed thresholds alone do not qualify. Grootjans (10.1016/j.radonc.2016.04.039 [61]) retains its historical full-text E1 exclusion. Current E1 decisions were adopted for Ferreira (10.1007/s00259-021-05303-5 [62]), Hatt (10.1007/s00259-011-1834-9 [63]; 10.1007/s00259-011-1755-7 [64]), Zhang (10.1007/s11548-012-0749-7 [65]), Zhou/Maquilan (10.1177/1533033820983804 [66]) and Alderuccio/LOTIS-2 (10.1158/1078-0432.CCR-23-1561 [67]). Blank historical reasons are not retrospectively relabelled. The Zhang report is not a Jiang-authored report. Possible conference/journal links for Jiang and Maquilan remain unconfirmed; the reports are not merged or counted as independent cohorts on that basis. Brunner P1435 is excluded as a conference abstract (E4), not for anatomy-only AI. MAA-Net retains E2 based on the abstract and selected main-text excerpts; its complete text was unavailable. These checks add no

included report and are not added as extra exclusions without record-level matching.

Following AI-assisted review, Sang Jun Byun independently reviewed the additional and changed revision materials, and Byungwook Choi made the final decisions. This verification did not constitute independent duplicate extraction or appraisal from scratch, or independent duplicate screening of all 2384 revision records. The absence of independent duplicate extraction and appraisal remains a methodological limitation.

The current synthesis contains 35 reports (13 Core, 22 Contextual) in 31 catalogue families: 32 from the reconciled original database branch, two from Google Scholar and one from the revision branch. Catalogue families do not establish independent patient cohorts. The 52 method-consistency flags overlap existing records (46 within the 2092 screening exclusions and six within the 292 follow-up records) and are not additional records. All 52 conference-record dispositions are E4; source-specific journal eligibility is considered separately. The 62 QUIPS units cover 33 reports; 37 have high confounding risk and 50 high statistical-analysis/reporting risk. The 119 model-assessment records and 23 aligned comparisons remain unchanged.

**S25 Targeted source verification after final integration**

Targeted verification of reviewer-suggested reports was completed using newly supplied full texts and available supplementary information. Ji et al. (10.1007/s00259-026-08008-9 [43]) describe manual primary-tumour delineation and Otsu habitat thresholding (printed p.6492), followed by prognostic modelling. This supports an E1 exclusion proposal under the existing learning-based measurement rule, without adding an exclusion to the historical denominator. The report was already cited as background literature. Its Table 2 compares response models, and Supplementary Table 5 reports progression analyses; these do not establish the incremental prognostic contribution of an AI-derived burden measure over MTV. Zuo et al. (10.1002/hed.27895 [68]) and Li et al. (10.1002/jmri.70492 [69]) concern clinical/MRI-based prediction rather than eligible AI-derived PET burden and remain outside the synthesis.

The supplied Maquilan conference abstract (2016, S51, abstract 113) confirms 86 patients treated at UT Southwestern during 2009–2012, manually seeded semiautomated PET segmentation, and downstream SVM prediction. Its correct DOI is 10.1016/j.ijrobp.2016.06.134 [70]; the DOI ending in .132 in the archived export identifies a different report. This is a proposed bibliographic correction in a separate verification record; the archived export and adopted E4 disposition are preserved. Its exact patient relationship to the 75-patient Zhou/Maquilan journal report remains unresolved. Jiang conference record EM26-L71396796 retains E4; the original conference PDF was not retrieved, and linkage to the related 2015

Zhang/Jiang journal report (10.1016/j.lungcan.2015.03.023 [71]) remains unconfirmed despite matching cohort characteristics. Brunner P1435 retains E4, with no confirmed journal companion. Learned anatomical masks contributing to PET burden are not rejected solely for being anatomical. MAA-Net (PII S1746809426017465; registered DOI 10.1016/j.bspc.2026.111192 [72]) retains its abstract/excerpt-based E2 disposition. Complete text and exact first online date remain unavailable; the November 2026 issue date and August DOI registration do not establish that date. Source limitations are not evidence of nonpublication.

These checks do not change the 35 included reports, 31 catalogue families, 2384 active revision records, original 101/69/32 full-text accounting, 62 QUIPS units from 33 reports, 119 PROBAST+AI records or 23 aligned comparisons. AI-assisted proposals and source checks are documented separately from the subsequent independent review by Sang Jun Byun and final decisions by Byungwook Choi. Web of Science, Scopus and IEEE Xplore are recorded as historical coverage limitations, not outstanding search tasks.
